# Targeting the NLRP3 inflammasome in kidney disease: molecular mechanisms, pathogenic roles, and emerging small-molecule therapeutics

**DOI:** 10.3389/fimmu.2025.1703560

**Published:** 2025-11-20

**Authors:** Yanfang Luo, Muyang Long, Xueqin Wu, Liuting Zeng

**Affiliations:** 1 Department of Nephrology, The Central Hospital of Shaoyang, Shaoyang, Hunan, China; 2 Department of Rheumatology and Clinical Immunology, Peking Union Medical College Hospital, Chinese Academy of Medical Sciences & Peking Union Medical College, National Clinical Research Center for Dermatologic and Immunologic Diseases (NCRC-DID), Key Laboratory of Rheumatology and Clinical Immunology, Ministry of Education, Beijing, China

**Keywords:** NLRP3 inflammasome, kidney disease pathogenesis, small-molecule inhibitors, pyroptosis, therapeutic targeting

## Abstract

Inflammatory responses represent a core pathological process driving the progression of both acute and chronic kidney diseases. As a key effector of the innate immune system, the NLRP3 inflammasome is widely activated in renal resident cellsand infiltrating immune cells, positioning it as a critical nexus linking metabolic dysregulation, cellular stress, and tissue injury. Accumulating preclinical and clinical evidence in recent years demonstrates that aberrant activation of the NLRP3 inflammasome directly promotes glomerular damage, tubulointerstitial inflammation, fibrosis, and vascular dysfunction through the release of IL-1β and IL-18 and the induction of pyroptosis, thereby contributing to the pathogenesis of diverse renal disorders including acute kidney injury (AKI), diabetic kidney disease (DKD), IgA nephropathy, lupus nephritis, and chronic renal fibrosis. This review systematically delineates the multilayered regulatory mechanisms of the NLRP3 inflammasome within the renal microenvironment—including upstream activating signals, downstream effector pathways, and crosstalk with autophagy, mitochondrial dynamics, and epigenetic regulation. We particularly focus on how disease-specific triggers in kidney pathologies such as hyperglycemia, uric acid, lipotoxicity, and ischemia reperfusion instrumentalize NLRP3 to drive irreversible renal injury. Critically, we provide a comprehensive evaluation of current advances in the development of small-molecule inhibitors targeting the NLRP3 inflammasome pathway, encompassing preclinical and clinical trial data for agents that directly modulate NLRP3 protein conformation, inhibit ASC oligomerization, block caspase-1 activity, or neutralize IL-1β. We further dissect the differential therapeutic efficacy, tissue selectivity, safety margins, and emerging resistance mechanisms of these inhibitors across distinct renal disease models, while highlighting key translational challenges—including the lack of validated biomarkers, difficulties in patient stratification, and inefficient renal-targeted drug delivery. This review aims to establish a systematic theoretical framework for mechanistic research into renal inflammatory diseases and to provide a target rationale and a clinical development roadmap for the design of next-generation precision anti-inflammatory therapies, thereby accelerating the translation of NLRP3-targeted interventions from bench to bedside for patients with kidney disease.

## Graphical Abstract

NLRP3 inflammasome and kidney diseases

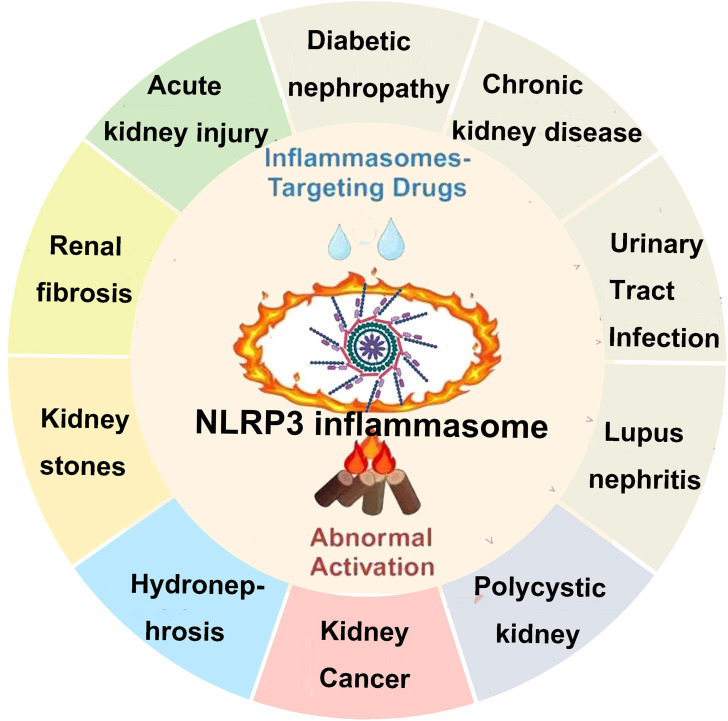

## 1 Introduction

**Figure 1:**
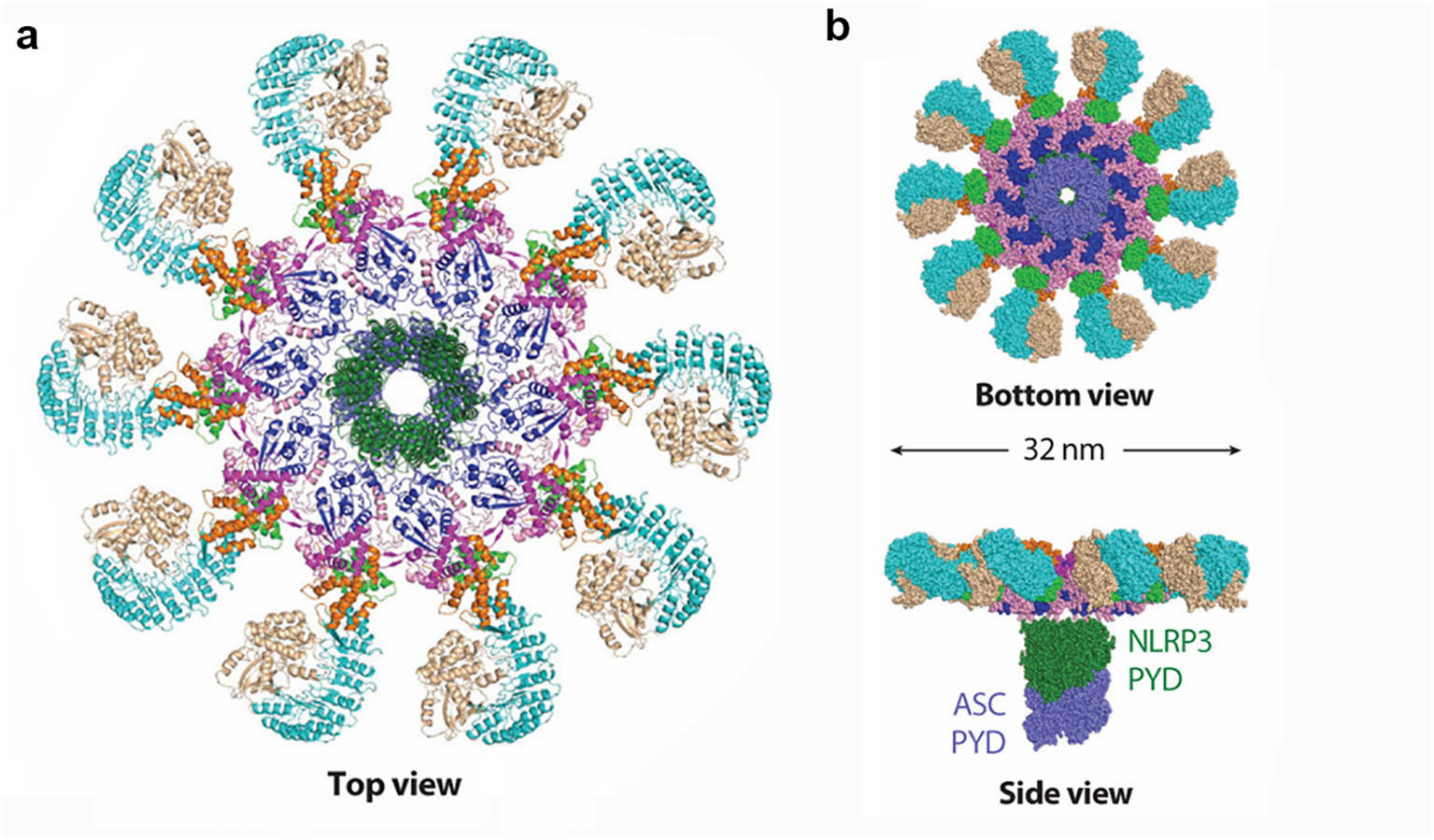
Structural details of the activated NLRP3 inflammasome disk. NLRP3 molecules are colored by domain. **(a)** Ribbon diagram of the activated NLRP3 inflammasome disk viewed from the top (PDB: 8EJ4). **(b)** Surface representation of the activated NLRP3 inflammasome disk viewed from the bottom and side, with the nucleating PYD–PYD filament formed by NLRP3 PYD (dark green) and ASC PYD (light purple) at the center of the disk (PDB: 8EJ4).

The incidence of kidney disease has been increasing year by year, especially chronic kidney disease (CKD), which has now become one of the major diseases threatening global public health. It is estimated that there are 850 million kidney disease patients worldwide (based on the current global population of approximately 7.2 billion, nearly 1 in 10 people are affected), with CKD causing approximately 2.4 million deaths annually. According to data from China, the prevalence of CKD among adults is 10.8%, with an estimated 120 million CKD patients across all age groups (1). Current research has found that the basic causes of CKD are numerous, including primary and secondary glomerulonephritis, DKD, hypertensive nephropathy, tubulointerstitial diseases, genetic diseases, and more (2). Pathological studies have shown that programmed cell death (apoptosis) plays a major role in AKI and its progression to CKD. This programmed cell death is a major pathological mechanism leading to renal unit loss and acute tubular necrosis (3). Relevant studies indicate that the levels of inflammatory markers are positively correlated with the occurrence and progression of CKD (4). Although many patients may not exhibit obvious clinical signs of inflammation, inflammatory factors can bind to damaged renal tissue cells and deposit in the kidney tissue, leading to excessive deposition of the extracellular matrix in the injured renal cells, thus promoting fibrosis progression (5). The presence of chronic low-grade inflammation in CKD patients is a reliable indicator of CKD prognosis and an independent risk factor affecting disease progression (6). The inflammatory response is a stress reaction that occurs when the body is exposed to external threats or environmental stresses. It has dual regulatory functions: on one hand, it can eliminate damaged or dead cells within the body, maintaining the body’s health; on the other hand, spontaneous inflammation and long-term chronic inflammation in the body can exacerbate the condition and lead to a series of complications (7, 8). Helicobacter pylori causing long-term gastric mucosal inflammation can lead to the development of gastric cancer (9). Long-term, chronic sterile inflammation in the synovial tissue of rheumatoid arthritis can lead to joint inflammation, pain, and even deformity. The novel coronavirus (SARS-CoV-2) can trigger the release of inflammatory factors in the body, leading to a cytokine storm, which causes multiorgan failure and death in patients (10). The inflammatory response is induced by various inflammasomes, and NLRP3 is a key regulatory protein. A comprehensive understanding of the role and regulatory mechanisms of NLRP3 in signaling pathways is of significant research guidance significance for disease prevention and drug discovery (11, 12). NLRP3 can be divided into three parts based on the protein’s structure and function: PYD, NACHT, and LRR. The PYD region located at the N-terminus can bind with the PYD structural domain of other proteins, forming a PYD–PYD complex to activate downstream reactions, playing a role in recruiting and linking. For example, binding with the C-terminal PYD region of ASC forms the NLRP3–ASC complex (13). The NACHT domain binds to ATP, hydrolyzing it into adenosine diphosphate (ADP) to release energy, which plays an important regulatory role in downstream proteins of NLRP3. The LRR region is rich in highly conserved leucine-rich repeat sequences and has a positive charge. Upon activation, it can form a NEK7–NLRP3 complex with NIMA-related kinase 7 (NEK7) through ion interactions and can be easily modified by ubiquitination, leading to self-inhibition of NLRP3 (11, 14) (**Figure 1**). Current research (15–17) shows that inflammation responses play a role in kidney diseases caused by various reasons. Inflammatory responses, mediated by inflammasomes as a central factor in sterile inflammatory reactions, contribute significantly to the progression of kidney diseases through pyroptosis. Therefore, interventions targeting inflammation based on the characteristics of pyroptosis are likely to offer excellent therapeutic effects in the prevention and treatment of various kidney diseases, bringing new targets to clinical diagnosis and treatment.

## 2 Regulation and inhibition of the classical activation pathway of NLRP3

### 2.1 Regulation mechanism of the NLRP3 classical signaling pathway

In resting phagocytes, NLRP3 is present at low levels and predominantly exists in a ubiquitinated, inactive, yet stable state (18). Canonical activation of the NLRP3 inflammasome requires two sequential signals. The priming signal is typically initiated by pathogen- or damage-associated molecular patterns—such as lipopolysaccharide (LPS)—which engage Toll-like receptor 4 (TLR4) to form a (TLR4/MD-2/LPS)_2_ hexameric complex with myeloid differentiation factor 2 (MD-2) (19). This activates the MyD88-dependent pathway involving IL-1 receptor-associated kinase 1 (IRAK1) and TNF receptor-associated factor 6 (TRAF6), ultimately promoting nuclear translocation of NF-κB and upregulating NLRP3 and pro-IL-1β expression. The second activation signal is provided by a diverse array of structurally unrelated stimuli—including viral RNA, fungal hyphae, extracellular ATP, hyaluronic acid, reactive oxygen species (ROS), uric acid crystals, β-amyloid proteins, and perturbations in transmembrane ion flux—many of which drive sterile inflammation relevant to kidney injury (19).

Upon stimulation, these triggers induce disintegration of the trans-Golgi network (TGN), generating dispersed vesicle-like structures termed disrupted TGN (dTGN). Phosphatidylinositol-4-phosphate (PtdIns4P), a negatively charged phospholipid enriched on dTGN membranes, recruits cytosolic NLRP3 via electrostatic interaction with its leucine-rich repeat (LRR) domain, facilitating its oligomerization (20). Following recruitment, NLRP3 undergoes activating posttranslational modifications such as deubiquitination and acetylation, whereas phosphorylation can suppress its activity. Notably, SIRT2 deacetylates NLRP3 and thereby inhibits assembly of the NLRP3/ASC/caspase-1 complex, exerting anti-inflammatory effects. The decline in SIRT2 content and activity with aging contributes significantly to immune dysregulation and the increased susceptibility to inflammatory diseases in the elderly (21).

Activated NLRP3 binds the C-terminus of NEK7 via its LRR domain to form the NLRP3–NEK7 complex, which nucleates the adaptor protein ASC through homotypic PYD–PYD interactions. ASC then recruits procaspase-1 via CARD–CARD binding, enabling its autocatalytic cleavage into active caspase-1. The resulting NLRP3 inflammasome complex cleaves pro-IL-1β and pro-IL-18 into their mature, bioactive forms for extracellular release and also cleaves gasdermin D (GSDMD). The N-terminal fragment of GSDMD forms plasma membrane pores that facilitate cytokine secretion and execute pyroptotic cell death (17). This cascade is tightly counterbalanced by endogenous inhibitors: PYD-only proteins (POPs), including POP1, POP2, and POP4, and CARD-only proteins (COPs), such as INCA, Iceberg, and caspase-12, which competitively disrupt PYD or CARD interactions, thereby preventing inflammasome assembly and limiting excessive inflammation (22).

### 2.2 Inhibition of NLRP3 activation at the transcriptional stage

Receptors on the outer membrane of phagocytic cells receiving stimulation from activating factors and NF-κB translocating into the nucleus to upregulate the expression of inflammatory proteins are key steps in the transcriptional activation of NLRP3. Z20 targets and binds to TLR4/MD-2, inhibiting the secretion of inflammatory factors and inflammatory responses, thereby effectively reducing organ damage induced by LPS and improving the survival rate of septic mouse models (23). T5342126 is a novel small-molecule TLR4 inhibitor that targets and binds to TLR4, preventing the formation of the TLR4–MD-2 complex, inhibiting TLR4 activity, and effectively enhancing the analgesic effect of morphine (19). Curcumin binds to the hydrophobic pocket of the MD-2 molecule, obstructing the formation of the TLR4/MD-2 complex and downregulating the activation of NF-κB (24). E5564 can inhibit the TLR4/NF-κB signaling pathway, reduce the activation of the NF-κB signaling pathway in macrophages caused by needle-like uric acid crystals in gout patients, and produce an effective anti-inflammatory effect (25). miR-233 can inhibit the activation of NF-κB by directly targeting the gene sequence of IRAK1, thereby producing an anti-inflammatory effect (26). The deubiquitinating enzyme A20 can recruit TNFR1 and cleave the Lys-63-linked polyubiquitin chains on it, leading to its deubiquitination and inactivation, thereby inhibiting the activation of NF-κB (27). BAY-117082 selectively and irreversibly inhibits IKK activity and exhibits significant anti-inflammatory activity in a mouse model of arrhythmogenic cardiomyopathy. Bortezomib can inhibit the ubiquitination of IκB subunits, downregulate NF-κB activity, reduce tumor volume in lung adenocarcinoma mice, and clinical studies have confirmed a significant improvement in survival rates for multiple myeloma patients treated with bortezomib (28).

### 2.3 Inhibition of NLRP3/ASC/caspase-1 complex formation

The formation of the NLRP3/ASC/caspase-1 complex can be inhibited by competitive binding using POPs and COPs, as well as by preventing protein deubiquitination, phosphorylation, and other activating actions to suppress complex formation and achieve anti-inflammatory effects. VX-765 and Ac-YVAD-cmk are both selective inhibitors of caspase-1. Experimental evidence has shown that VX-765 can significantly inhibit polyphyllin VI-induced activation of NLRP3 inflammasomes and cell death (29). Ac-YVAD-cmk can improve cognitive function in stroke mice through this pathway and restore hippocampal volume (30). b-AP15 targets the DUBs UCH37 and USP14 subtypes, inhibiting LPS-induced IL-1β secretion, reducing cell death caused by nigericin. Similarly, WP1130 targets four DUBs subtypes and exhibits activity similar to b-AP15. Research suggests that they can also inhibit the caspase-1 pathway by preventing the cleavage of the p10 subunit of caspase-1, thereby hindering complex formation (31). Bile acid receptor (TGR5) agonists such as betulinic acid, INT-777, and LCA can activate the PKA kinase by upregulating the TGR5/cAMP/PKA pathway. This introduction of a phosphate group at this juncture deactivates NLRP3, thereby exerting an anti-inflammatory effect (32). MCC950 has been experimentally proven through drug affinity, target stability, and other tests. It targets a small segment on the NACHT domain of the NLRP3 protein, known as the Walker B region, inducing a conformational change that inhibits NLRP3 activity. It exhibits significant inhibitory effects on inflammation triggered by LPS, Mycobacterium tuberculosis, and other pathogens (33).

## 3 Other regulatory pathways of NLRP3

### 3.1 Regulation of the non-classical activation pathway of NLRP3

**Figure 2:**
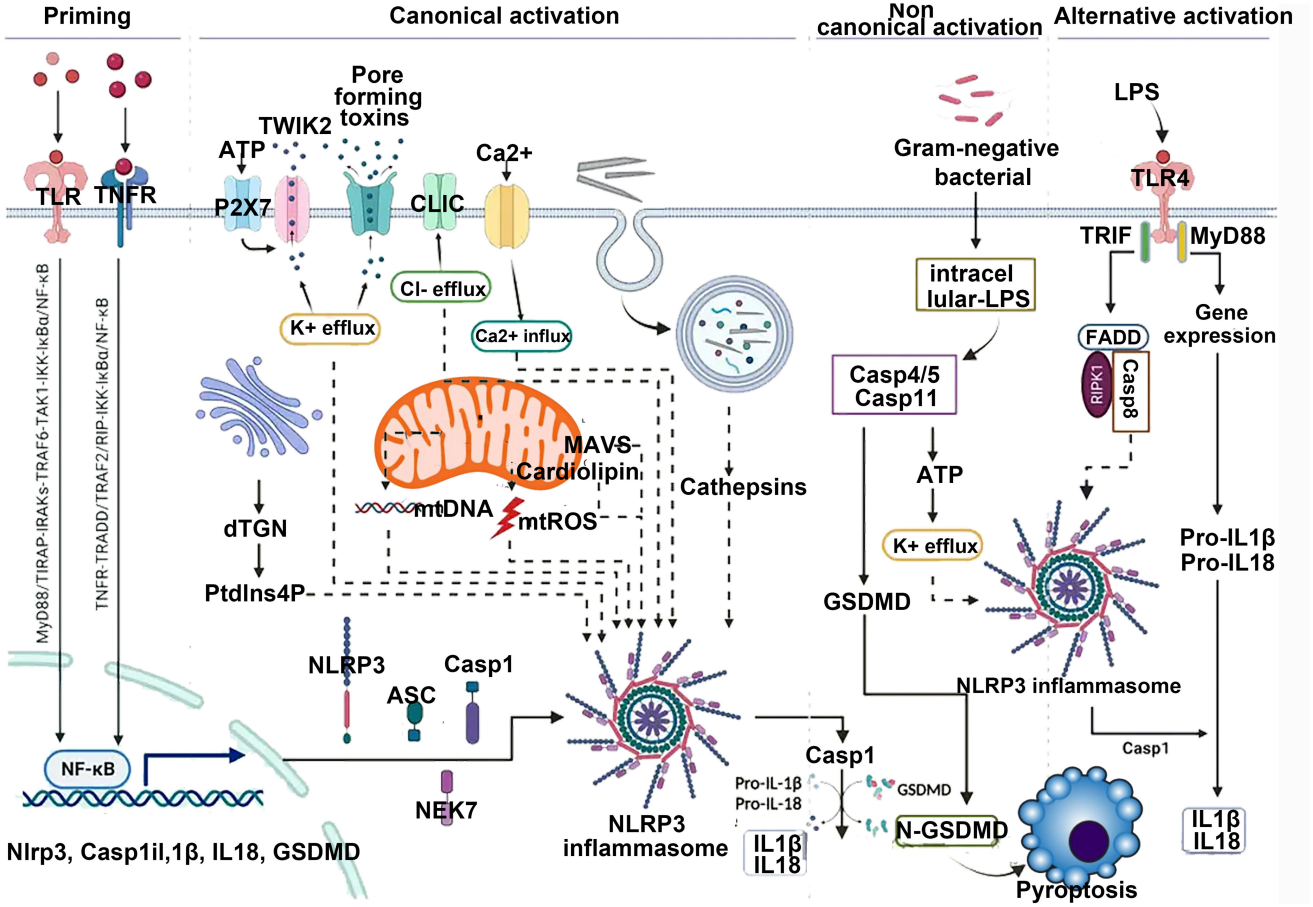
Canonical activation of NLRP3 proceeds via a two-step pathway. Step 1: Signal 1 (also termed “priming”), acts through cell surface receptors such as TNF-R, TLR, or IL-1R. This pathway induces priming at both the transcriptional level (NLRP3 itself or, more prominently, precursor forms of IL-1 family cytokines) and the posttranslational level (NLRP3 and other pathway components). This includes removal and addition of modifications that place NLRP3 into a primed or “armed” state. Subsequently, signal 2 can trigger NLRP3 activation. This may involve multiple agonists, many of which act by inducing intracellular potassium efflux. These potassium-dependent agonists include pore-forming toxins (e.g., nigericin or LukAB), amyloid proteins, ion channels, and lysosome-disrupting agents. Additionally, potassium-independent agonists exist, which appear to act via mitochondria and/or mitochondria-derived activators, such as oxidized mitochondrial DNA or cardiolipin. Through currently unknown molecular steps, both potassium-dependent and potassium-independent stimuli converge on NLRP3 and promote its activation, involving conformational changes that enable initial binding to ASC and subsequently to caspase-1. This may occur via one of two parallel pathways and may involve the adaptor protein NEK7. Within the fully assembled inflammasome, caspase-1 processes IL-1 family cytokines as well as other substrates such as GSDMD. Particularly in its cleaved form, GSDMD forms pores that facilitate the release of IL-1 and other alarmins but also lead to cell death in the form of pyroptosis.

As shown in region C of **Figure 2**, the three proteins Caspase-4, 5, and 11 can be activated by intracellular bacterial endotoxins, leading to the release of IL-1β and IL-18 by the NLRP3/ASC/caspase-1 complex. Additionally, these three proteins can directly act on GSDMD (34), producing effects similar to the classical activation pathway. Emricasan has been shown to inhibit liver inflammation and fibrosis, effectively alleviating alcohol-induced cirrhosis (35).

### 3.2 The effect and inhibition of transmembrane ion flux on NLRP3

Experimental data comparing the content of intracellular protein complexes indicate that K+ efflux can drive the aggregation of NLRP3, whereas Cl− efflux promotes the aggregation of ASC (36). As shown in region D of **Figure 2**, inhibiting ion flux can play a suppressive role in the activation of NLRP3. Some NLRP3 activators like imiquimod and CL097 activate NLRP3 by inducing K+ efflux (37). NPBB is a Cl^−^ channel blocker that can maintain low levels of intracellular Cl^−^, thereby inhibiting the activation of NLRP3; the antiplatelet drug ticagrelor acts on Cl^−^ channels by inducing the degradation of channel proteins and inhibiting the membrane localization of chloride channel proteins to inhibit Cl^−^ efflux achieving the effect of inhibiting the activation of NLRP3 (38).

### 3.3 The effect and inhibition of endoplasmic reticulum-related proteins on NLRP3

SREBP2 and SCAP, located on the endoplasmic reticulum, form an NLRP3/SREBP2/SCAP ternary complex that “transports” NLRP3 from the endoplasmic reticulum to the Golgi membrane, optimizing the assembly process of the inflammasome. The nitrofuran group of ESI targets the endoplasmic reticulum, disrupting its homeostasis. By influencing the synthesis of caspase-1 and reducing the secretion of IL-1β, an anticancer effect similar to bortezomib is generated (31). Terbutaline, fatostatin, and 25-HC can inhibit the SREBP2/SCAP pathway on the endoplasmic reticulum at the cellular level and in mouse experiments, affecting the assembly of NLRP3 and inhibiting the inflammatory response induced by LPS (39).

### 3.4 The effect and inhibition of mitochondria and related proteins on NLRP3

In the resting state, NLRP3 is located in the endoplasmic reticulum, whereas ASC is dispersed in the cytoplasm. As shown in area D of **Figure 2**, upon external stimulation, the MAVS protein located on the mitochondrial membrane interacts with the N-terminus of NLRP3, recruiting NLRP3 and ASC together, facilitating their activation. MAVS is an important protein for NLRP3 activation. Knocking out the MAVS gene in mice significantly inhibits the increase in IL-1β levels induced by LPS. MicroRNA-33/33* is an important regulatory factor for cholesterol homeostasis, which can silence AMPK posttranscriptionally, disrupt mitochondrial homeostasis, reduce MAVS activity, and hinder its recruitment of NLRP3 and ASC (40). Moreover, mitochondrial damage leads to the release of ROS, activating the NLRP3 inflammasome. Mitochondrial autophagy can suppress the activation of the inflammasome. Choline kinase (ChoK) inhibitors can promote mitochondrial autophagy by halting choline intake. After treatment with RSM932A, LPS-induced macrophages exhibit significant suppression of inflammatory effects, effectively alleviating symptoms in Muckle–Well syndrome in mice (41).

### 3.5 The effect and inhibition of inflammatory cytokines on NLRP3

Inflammatory cytokines are endogenous substances secreted into the extracellular space by immune cells upon activation, which can exert activating or inhibitory effects on surrounding other immune cells. Canakinumab is a fully human monoclonal IgG1/k antibody used to treat various IL-1-mediated inflammatory diseases. It selectively binds to free IL-1β, blocking its interaction with IL-1R, thereby inhibiting IL-1β activity. In clinical trials across various disease models, canakinumab has demonstrated significant anti-inflammatory effects in conditions such as cryopyrin-associated periodic syndromes (CAPS), systemic juvenile idiopathic arthritis (sJIA), and tumor necrosis factor receptor-associated periodic syndrome (TRAPS) (42).

TNF-α monoclonal antibodies such as etanercept, adalimumab, and infliximab can bind to free extracellular TNF-α, inhibiting the activation of NLRP3. Clinical evidence has shown significant therapeutic effects of adalimumab in immune-mediated chronic diseases like psoriasis and Crohn’s disease (43). IL-10 targets to reduce the translation expression of NLRP3, demonstrating a significant inhibitory effect on inflammation. However, IL-10 has a short half-life *in vivo* and is prone to inactivation. A PEGylated IL-10 inhibitor, pegilodecakin, extends the stimulation time of IL-10 receptors, exerting a significant anti-inflammatory effect. Clinical trials evaluating the safety and effectiveness of combination therapy with anti-PD-1 monoclonal antibody inhibitors are currently underway (44).

**Figure 3:**
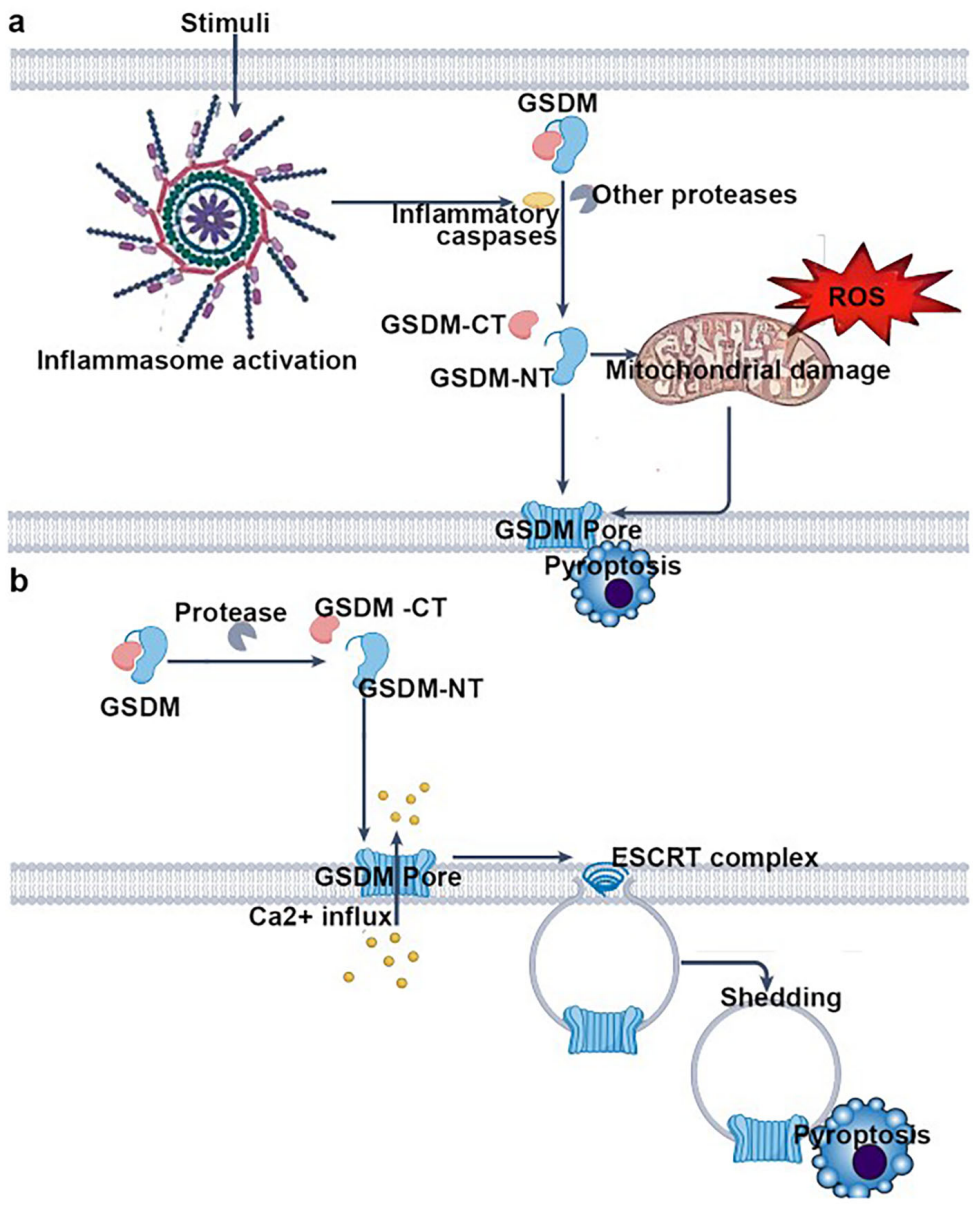
Key steps of pyroptosis mediated by gasdermin (GSDM) family proteins, organized into two major pathways: pathway 1—inflammasome-dependent GSDM activation and pyroptosis; inflammasome activation—stimuli trigger the assembly and activation of inflammasomes. GSDM cleavage: Activated inflammasomes recruit and activate inflammatory caspases (or other proteases), which cleave gasdermin (GSDM) into its C-terminal (GSDM-CT) and N-terminal (GSDM-NT) domains. Mitochondrial damage and pore formation: GSDM-NT translocates to the plasma membrane. Concurrently, reactive oxygen species (ROS) induce mitochondrial damage, further promoting GSDM-NT–mediated pore (GSDM pore) formation in the plasma membrane. Pyroptotic cell death: Pore formation disrupts osmotic balance, leading to cellular swelling, release of intracellular contents, and ultimately pyroptosis. Pathway 2: Protease-dependent GSDM activation and ESCRT-mediated pore repair–associated pyroptosis; GSDM cleavage: specific proteases directly cleave gasdermin (GSDM), generating GSDM-CT and GSDM-NT. Pore formation and Ca²^+^ influx: GSDM-NT forms pores in the plasma membrane, triggering Ca²^+^ influx. ESCRT-mediated pore repair and shedding: The influx of Ca²^+^ recruits the ESCRT (endosomal sorting complex required for transport) machinery, which attempts to repair the pores. During this repair process, pore-containing membrane regions are shed (“shedding”), yet pyroptosis still ensues. In summary, this figure comprehensively depicts the core mechanisms of pyroptosis involving GSDM cleavage, pore formation, and ESCRT complex–mediated modulation, highlighting the central paradigm of pyroptosis: “pore formation → (attempted repair) → cell death”.

In summary, the activation factors of NLRP3 are diverse, and this pathway is closely associated with the occurrence and progression of many challenging diseases. Exploring novel NLRP3 inhibitors holds great promise for a wide range of applications. Inhibiting the activity of NLRP3 inflammasomes can significantly alleviate AKI and septic myocarditis damage (45, 46). In recent years, research on the regulatory mechanisms of the NLRP3 inflammasome has become a frontier hotspot. The small-molecule inhibitor MCC950 has been shown to directly inhibit the activity of NLRP3, attracting significant attention. A deep understanding of the physiological and pathological processes of inflammation, along with the exploration of new targets and highly selective inhibitors based on its activation pathways, can provide a fresh approach to treating major inflammatory-related diseases. The detailed pathways of NLRP3 activation are illustrated in **Figures 2**, **3** below.

### 3.6 The mechanism of NLRP3 inflammasome activation in the kidneys

NF-κB/NLRP3 is one of the important pathways for NLRP3 inflammasome activation. ROS-induced NLRP3 inflammasome activation promotes the formation of calcium oxalate kidney stones (47). Research has shown that inhibiting the NLRP3 inflammasome in diabetic nephropathy improves podocyte injury by suppressing lipid accumulation (48). The potential renal protective effect of resveratrol in a rat model of gouty nephropathy may involve inhibiting the NF-κB signaling pathway, subsequently suppressing NLRP3 activation to block the recruitment of Caspase-1 for IL-1β and IL-18, reducing their secretion, inhibiting the occurrence of pyroptosis, a type of programmed cell death in renal cells’ initial stages, thus potentially reversing the inflammatory damage in the kidney tissue of rats with gouty nephropathy. Research suggests that the potential renal protective effect of resveratrol in a rat model of gouty nephropathy may involve inhibiting the NF-κB signaling pathway, subsequently suppressing NLRP3 activation to block the recruitment of caspase-1 for IL-1β and IL-18, reducing their secretion, inhibiting the initiation of programmed cell death in renal cells, particularly the occurrence of pyroptosis at the initial stage, thus reversing the inflammatory damage in the kidney tissue of rats with gouty nephropathy (49). Epimedium glycoside and magnolol may alleviate kidney damage in patients with lupus nephritis (LN) by modulating the NF-κB/NLRP3 pathway (50, 51). Research has shown that the purinergic receptor P2X7 plays a significant role in the activation of the NLRP3 inflammasome (52). In the lupus nephritis mouse model, the study found a significant increase in inflammatory molecules in the P2X7/NLRP3 signaling pathway. Inhibiting P2X7 can suppress the assembly of NLRP3–ASC–caspase-1, indicating the crucial role of the NLRP3 inflammasome in LN. A recent study found that P2X4 in an ischemia–reperfusion (I/R)-induced AKI mouse model triggers kidney inflammation and renal cell apoptosis by activating the NLRP3 inflammasome (53). Conversely, P2X4 deficiency can prevent ischemic AKI, reduce renal tubular necrosis, alleviate renal cell apoptosis, and decrease neutrophil infiltration in the kidney. High glucose, lipopolysaccharides, and oxidative stress can promote the assembly and activation of the NLRP3 inflammasome (54, 55). ROS-thioredoxin-interacting protein (TXNIP) is another important molecule in the process of NLRP3 inflammasome activation. Gao et al. and Wang et al. found that in a high-glucose environment, TXNIP activates the reduced form of nicotinamide adenine dinucleotide phosphate (NADPH) oxidase, leading to NLRP3 inflammasome activation in podocytes, subsequently causing podocyte damage (56, 57). Wen et al. found that inhibiting mitochondrial ROS production can suppress the colocalization of NLRP3 and TXNIP, as well as the activation of the NLRP3 inflammasome. Additionally, TXNIP siRNA significantly inhibited the activation of the NLRP3 inflammasome in a mouse model of I/R injury. This study indicates that the mROS-TXNIP-NLRP3 pathway is a key signaling cascade in I/R-induced AKI, providing a new avenue for gene therapy targeting the NLRP3 inflammasome signaling pathway (58).

### 3.7 The mechanism of NLRP3 activation in the kidney independent of inflammasome

Apart from the NLRP3 inflammasome, NLRP3 also exerts its function independently of the inflammasome in the kidney. Wang et al. reported that NLRP3, independent of the inflammasome, directly promotes transforming growth factor-β (TGF-β) signaling and R-Smad activation, thereby inducing epithelial–mesenchymal transition (59). The fibrotic signals induced by TGF-β can be attenuated in fibroblasts lacking NLRP3 (60). NLRP3 can also form a complex with ASC and caspase-8 in mitochondria, regulating cell apoptosis in kidney and intestinal epithelium (61). During the apoptosis process, mitochondrial antiviral signaling protein (MAVS) can associate with and activate caspase-8 in mitochondria (62). Kim et al. found that under hypoxic conditions, NLRP3 in renal tubular cells relocalized from the cytoplasm to the mitochondria and interacted with MAVS (63). The absence of NLRP3 or MAVS during hypoxia reduced mitochondrial ROS production and mitochondrial membrane depolarization, thereby protecting the kidney from injury. Therefore, NLRP3 can act independently of the inflammasome, and further research is needed to elucidate its mechanisms.

## 4 Inflammasome and kidney diseases

### 4.1 Inflammasome and CKD

Inflammatory responses significantly promote the progression of CKD by activating the NLRP3–ASC–caspase-1 axis to induce and release inflammatory cytokines like IL-1β and IL-18, which play pivotal roles in the onset and advancement of CKD (16). Studies have revealed that the NLRP3 inflammasome signaling pathway is present in myocytes and possesses biological activity. Notably, the TLR4/NLRP3 inflammasome pathway contributes to the promotion of skeletal muscle inflammation in patients with CKD (64). Anti-inflammatory diets hold potential for the prevention of CKD (65). NLRP3 is involved in the occurrence and development of kidney disease, whether in glomerular cells, tubular cells, interstitial cells, or infiltrating inflammatory cells (66). In lupus model mice, activation of the NLRP3 inflammasome was observed in podocytes, leading to renal tissue damage, podocyte foot process disruption, and the manifestation of proteinuria (67). In diabetic nephropathy mice, significant expression of NLRP3 and caspase-1 is observed in glomerular endothelial cells and podocytes. When NLRP3 or caspase-1 is knocked out in mice, there is a significant reduction in urinary protein levels (68). Mice with NLRP3 knockout exhibit a noticeable attenuation in foot cell damage induced by elevated homocysteine and in the progression of glomerulosclerosis (16). In an obese-related foot cell injury mouse model, knocking out ASC results in a decrease in foot cell NLRP3 inflammasome activation. This leads to a reduction in urinary protein levels and a mitigation of glomerulosclerosis (69). The NLRP3 inflammasome is involved in renal interstitial damage. Ikeda et al. found a significant increase in the expression of NLRP3, ASC, and caspase-1, along with increased secretion of mature IL-1β in mice with renal tubulointerstitial injury induced by albumin overload (70). This led to a pronounced exacerbation of renal tubulointerstitial damage. In a unilateral ureteral obstruction (UUO)-induced renal tubulointerstitial inflammation model, NLRP3 knockout mice exhibit significantly reduced renal tubular injury and interstitial fibrosis compared with wild-type mice (71). In a mouse model of renal tubular injury induced by albumin overload, the activation of the NLRP3/caspase-1/inflammatory cytokine cascade was observed, leading to cell apoptosis and phenotypic changes. Severe tubular structural damage and renal tubular cell apoptosis were also evident (72). This indicates the involvement of NLRP3 in renal tubular injury. Hyperuricemia is a significant risk factor for cardiovascular and kidney diseases. When human proximal tubular epithelial cells are stimulated with uric acid *in vitro*, there is a marked increase in the expression of NLRP3 and the activation of IL-1β (73). Similarly, when human mesangial cells are stimulated with high glucose *in vitro*, the expression of NLRP3, caspase-1, and IL-1β increases in a time-dependent manner (74). In addition to animal experiments and *in vitro* studies, the NLRP3 inflammasome also plays a crucial role in the occurrence and development of kidney diseases in humans. In human renal biopsy tissues, including IgA nephropathy, lupus nephritis, minimal change disease, hypertensive nephropathy, and secondary focal segmental glomerulosclerosis, significantly increased expression of NLRP3 mRNA has been detected compared with normal tissues. This elevated expression is positively correlated with kidney function impairment, suggesting that NLRP3 may be involved in the pathogenesis of CKD (71). The expression of NLRP3 has been detected in podocytes of patients with lupus nephritis, along with an increase in urine protein levels. This finding indicates a relationship between the activation of NLRP3, podocyte damage, and the formation of proteinuria (67). In patients with mesangial proliferative glomerulonephritis, an increased expression and secretion of NLRP3, caspase-1, IL-1β, and IL-18 have been observed in the renal tubular epithelial cells. This is accompanied by tubular epithelial cell degeneration, tubular atrophy, inflammatory cell infiltration, and inflammatory cell expression of the mentioned factors in the renal interstitium (75). Granata et al. found increased gene expression of NLRP3 inflammasome components and pro-inflammatory cytokines in peripheral blood mononuclear cells of CKD patients undergoing hemodialysis (76). The levels of caspase-1, IL-1β, and IL-18 were significantly higher than those in the healthy control group. This suggests the involvement of the NLRP3–ASC–caspase-1 axis in the occurrence and progression of kidney disease. The study by Lichtnekert et al. demonstrates that in a model of anti-glomerular basement membrane crescentic glomerulonephritis, endogenous glomerular cells cannot induce glomerulonephritis through the NLRP3–ASC–caspase-1 axis. However, dendritic cells present in the renal interstitium can activate this axis to secrete IL-1β, indicating that this process is independent of the NLRP3 inflammasome and does not rely on ASC-mediated caspase-1 activation (66). Another study demonstrates that in a model of serum-induced nephrotoxic nephritis, knockout mice lacking the NLRP3 and ASC genes exhibit significantly reduced glomerular damage and related inflammatory responses compared with the wild-type mice. In ASC knockout mice, a decrease in the secretion of active IL-1β is observed, whereas no changes are observed in NLRP3 knockout mice. This indicates the involvement of another potential molecular mechanism independent of the NLRP3 inflammasome. This may be related to the release of high-mobility group protein 1 in an NLRP3-mediated manner during glomerular nephritis (77), highlighting the complexity of NLRP3’s role in kidney diseases.

#### 4.1.1 Role and mechanism of the NLRP3 inflammasome in renal fibrosis

**Figure 4:**
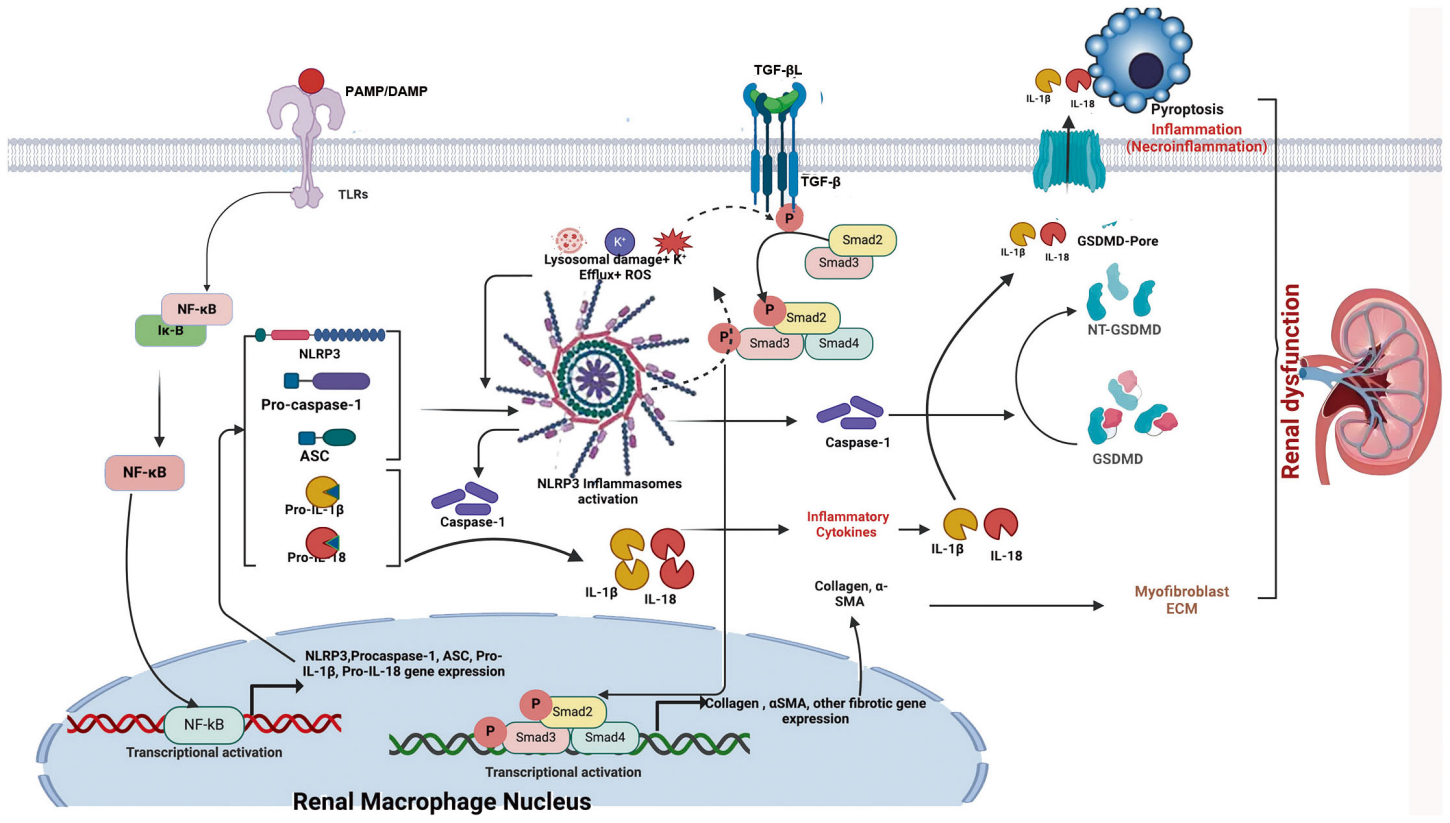
Schematic illustration of NLRP3 inflammasome- and TGF-β-induced renal fibrosis. When danger signals such as damage-associated molecular patterns (DAMPs) and pathogen-associated molecular patterns (PAMPs) bind to Toll-like receptors (TLRs) on renal macrophages/dendritic cells, transcriptional activation of the NF-κB signaling pathway is triggered. This activation leads to increased expression of inflammasome-associated components, including NLRP3, ASC, pro-caspase-1, pro-IL-1β, and pro-IL-18. Subsequently, events such as potassium efflux (K^+^), reactive oxygen species (ROS) generation, and lysosomal damage are induced. These events lead to NLRP3 activation and oligomerization, which in turn recruits ASC and pro-caspase-1 to form the inflammasome complex, ultimately converting pro-caspase-1 into biologically active caspase-1. Activated caspase-1 cleaves pro-IL-1β and pro-IL-18 into their mature inflammatory cytokines, IL-1β and IL-18, thereby mediating inflammation. In addition, activated caspase-1 cleaves GSDMD into NT-GSDMD, inducing pore formation in the plasma membrane and mediating pyroptosis, a regulated form of necrotic cell death (necroinflammation). Simultaneously, TGF-β binds to its receptor (TGF-βR); the activated NLRP3 inflammasome induces ROS generation, which enhances phosphorylation of regulatory Smad proteins (Smad2, Smad3, and Smad4), thereby promoting their transcriptional activation. This activation drives expression of fibrosis-related genes, such as collagen and α-smooth muscle actin. Crosstalk between the NF-κB/NLRP3/IL-1β/IL-18 axis and the TGF-β/Smad signaling pathway may contribute to the development of renal fibrosis and associated injury.

Renal fibrosis serves as a pivotal mechanism and shared pathway in the progression of CKD, culminating in progressive renal function decline and eventual end-stage renal failure (78). Adenine diet and UUO activate the NLRP3 inflammasome through ROS (79, 80). Recent reports further reveal NLRP3 inflammasome-dependent NF-κB activation following subtotal nephrectomy (81). The NLRP3 inflammasome drives renal fibrosis by activating T cells via the IL-23/IL-17 axis (79). Notably, MCC950 administration concurrent with adenine diet initiation attenuates renal fibrosis by inhibiting NLRP3 inflammasome activation, whereas delayed MCC950 treatment on day 7 of the diet fails to confer protection (80), underscoring the therapeutic necessity of early intervention to suppress inflammation and fibrosis progression. Endothelial cells (ECs) have also been implicated in renal fibrosis. In primary tubular epithelial cells (TECs) isolated from mouse kidneys, TGF-β induces NLRP3 upregulation. NLRP3 subsequently promotes TEC epithelial-to-mesenchymal transition (EMT) through Smad2/3 phosphorylation, leading to myofibroblast (MF) differentiation and elevated expression of α-smooth muscle actin (α-SMA) and matrix metalloproteinase 9 (MMP9). Conversely, TGF-β treatment of TECs from NLPR3−/− mice results in reduced Smad2/3 phosphorylation and diminished α-SMA/MMP9 expression, confirming that NLRP3 facilitates TEC-driven renal fibrosis via the TGF-β/Smad pathway (59). Emerging evidence highlights the NLRP3 inflammasome as a central mediator of DKD progression. Its activation in podocytes not only exacerbates glomerular inflammation but also promotes advanced glomerulosclerosis, establishing NLRP3 as a key inducer of renal fibrosis (82–84). Podocytes are highly specialized glomerular epithelial cells that are crucial for maintaining the integrity of the filtration barrier and particularly susceptible to metabolic stress. Notably, the therapeutic potential of targeting and inhibiting inflammasomes to regulate podocyte metabolism can exert a favorable renoprotective effect (85). In a multiple low-dose streptozotocin-induced diabetic mouse model, genetic NLRP3 inhibition mitigates oxidative stress, attenuates renal inflammation and fibrosis, and improves renal function (81). Beyond pathogen- or endotoxin-derived stimuli, mitochondrial ROS generation (86, 87) and lysosomal membrane destabilization (88) are recognized as critical triggers of NLRP3 inflammasome activation. Recent studies further demonstrate that PIPK3 modulates pyroptosis by stimulating NLRP3 inflammasome signaling (89). Collectively, these findings emphasize the therapeutic potential of targeting oxidative stress-mediated pyroptosis to prevent DKD driven by inflammasome hyperactivation. NLRP3 inflammasome-induced renal fibrosis is detailed in **Figure 4** below.

Extracellular calcium initiates signal transduction via the calcium-sensing receptor, effectively activating the NLRP3 inflammasome and inducing renal fibrosis (90). Schmidt-Lauber et al. also demonstrated that NLRP3 inflammasome activation promotes IL-1β secretion and renal fibrosis in a mouse model of contrast-induced nephropathy (91). *In vitro* experiments further indicate that IL-1β can drive the progression of CKD and induce the transformation of renal tubular epithelial cells into fibroblasts (92). Knockout of the NLRP3 gene preserves mitochondrial morphology in mouse renal tubules, ameliorates CKD-associated hypertension and proteinuria, and mitigates renal fibrosis (93, 94). In summary, the NLRP3 inflammasome mediates renal fibrosis through oxidative stress and inflammatory pathways (95). Additionally, NLRP3 can independently mediate renal inflammation, injury, and fibrosis outside of its role in the inflammasome complex.

#### 4.1.2 Role and mechanism of the NLRP3 inflammasome in DKD

The latest statistics from the International Diabetes Federation indicate that the global prevalence of diabetes has reached approximately 537 million individuals, with an adult incidence rate of 1 in 10. It is projected that by 2045, the total number of people with diabetes will increase to approximately 783 million, with an estimated adult incidence rate of 1 in 8 (96). With advancing research, the critical role of pyroptosis in the development and progression of DKD has been established. DKD is one of the most common causes of CKD. Under diabetic conditions, increased renal glucose load contributes to microvascular damage through elevated ROS, activation of the polyol pathway, and upregulation of injury mediators. Abnormal accumulation of ROS activates a cascade of signaling molecules, further upregulating injury mediators and exacerbating renal damage (97). Glomerular hyperperfusion, hyperpressure, and hyperfiltration are key factors in DKD. Increased extracellular matrix synthesis, glomerular fibrosis, tubular basement membrane disruption, and interstitial infiltration mediate tubulointerstitial fibrosis, gradually progressing to DKD. Among these factors, the role of inflammation has gained increasing recognition, with the NLRP3 inflammasome emerging as a key focal point of research. Specifically, chronic low-grade inflammation, which is primarily mediated through the IL-6 and NLRP3 inflammasome signaling pathways, contributes to the pathogenesis of diabetic kidney disease (98). NLRP3 mRNA levels are elevated in the kidneys of patients with type 2 diabetes and are even higher in those with diabetic nephropathy (DN) (99). Another study also found increased expression of the NLRP3 inflammasome in the renal tubules of diabetic patients with tubulointerstitial injury (68). Activation of the NLRP3 inflammasome has been observed in glomerular endothelial cells and podocytes in mouse models of DN (68). Activation of the NLRP3 inflammasome induces the production of pro-inflammatory cytokines and further promotes insulin resistance in patients with DN (100). Conversely, knockout or inhibition of NLRP3 reduces diabetic kidney injury (68). The activation of the NLRP3 inflammasome during DN involves multiple pathways, including the nuclear factor erythroid 2-related factor 2 (Nrf2) pathway (101), the ROS/TXNIP pathway (102), the NF-κB pathway, and the P2X7/NLRP3 pathway (103). Additionally, autophagy can suppress the activation of the NLRP3 inflammasome (104). In a rat model of DN, mitophagy alleviates systemic inflammatory responses and further damage by modulating the M1/M2 macrophage balance, maintaining homeostasis (105). Recent studies (106) have shown that NLRP3 mediates renal damage in a mouse model of DN by inhibiting podocyte autophagy.

Pyroptosis primarily occurs when inflammasomes, such as the nucleotide-binding oligomerization domain-like receptor protein 3 (NLRP3) inflammasome, are stimulated by inflammatory factors or pathogens to form complexes. This process further activates caspase-1, which cleaves downstream GSDMD into a 242-amino-acid N-terminal domain (GSDMD-N) and a 199-amino-acid C-terminal domain (GSDMD-C). GSDMD-N forms pores in the cell membrane, disrupting intracellular and extracellular homeostasis, leading to cell swelling, rupture, and the release of cellular contents and inflammatory cytokines such as interleukin (IL)-1β and IL-18, causing necrosis and intense inflammatory responses (107, 108). Caspase-1 is a key factor in initiating the canonical pyroptosis signaling pathway (109). The GSDMD protein is cleaved into GSDMD-N and GSDMD-C by active Caspase-1, with GSDMD-N forming pores in the cell membrane, leading to cell swelling, rupture, and necrosis accompanied by strong inflammatory responses (110). Studies have reported that silencing caspase-1 in a diabetic mouse model blocks inflammasome stimulation and protects against the progression of DKD (111). Evidence suggests that activation of the caspase-4/5/11 pathway contributes to various diseases, including inflammatory disorders, severe diabetic complications, and neurodegenerative diseases (112, 113). Caspase-11, a critical protein in the canonical pyroptosis pathway, suppresses the release of inflammatory cytokines when silenced, improving glomerular filtration function and podocyte morphology (114). Caspase-4, the human homolog of caspase-11, shows significantly increased protein expression in podocytes exposed to high-glucose environments. Studies indicate that high glucose promotes the expression of caspase-4 mRNA and protein in podocytes, and ELISA results show that high glucose increases IL-1β concentrations. Silencing caspase-4 via siRNA effectively suppresses the elevation of IL-1β levels in podocytes under high-glucose stimulation (115). Traditionally, caspase-3 activation was thought to induce apoptotic cell death; however, new evidence suggests that caspase-3 activation may also lead to pyroptotic cell death (116). Other studies report that, in addition to caspase-1, activation of caspase-3 and caspase-7 can trigger pyroptosis (117). Activation of caspase-8 can further activate the NLRP3 inflammasome, suggesting that caspase-8 may also be a key mediator of pyroptotic cell death (118). As research advances, an increasing number of caspase family members and their inhibitors have been identified, providing new insights and directions for studying pyroptosis-related signaling pathways and their potential roles in promoting DKD. Caspase inhibitors hold promise as novel therapeutic targets for treating DKD. A study found that high-glucose treatment significantly increases GSDMD mRNA and protein expression in podocytes, and silencing GSDMD suppresses mitochondrial ROS generation, indicating that GSDMD-dependent pyroptosis promotes renal inflammation and is a critical factor in the pathogenesis of DKD (119). Increased expression of caspase-4/11 and GSDMD-N proteins has been observed in podocytes under high-glucose conditions. These findings highlight the indispensable role of GSDMD, a key downstream substrate of pyroptosis, in this process. GSDMD inhibitors may play a crucial role in suppressing pyroptotic cell death, making them promising candidates for fundamentally preventing the onset of DKD.

##### 4.1.2.1 NLRP3 inflammasome and glomerular damage

The glomerular capillary wall is composed of endothelial cells, the basement membrane, and visceral epithelial cells (podocytes), surrounded by mesangial cells and matrix. Li Fang et al. detected the expression of caspase-1, IL-1β, and IL-18 in DN renal tubules, which positively correlated with the severity of proteinuria. In the same specimens, the expression of inflammatory factors was higher in renal tubules than in glomeruli (120). Hong Feng et al. were the first to demonstrate that high glucose induces the expression and activation of NLRP3 and pro-caspase-1 in mesangial cells, leading to the release of IL-18, increased glomerular and mesangial area, and enhanced collagen accumulation in the kidney (121). Chenlin Gao et al. found that receptor-interacting protein kinase 2 (RIPK2)-mediated podocyte autophagy negatively regulates ROS-NLRP3 inflammasome signaling under high-glucose conditions. High glucose activates autophagy in the short term but suppresses it over prolonged periods. Activation of NLRP3 inhibits podocyte autophagy, weakening the protective effects mediated by autophagy and exacerbating podocyte damage (122). Chun Zhang et al. observed foot process effacement, loss of slit diaphragm molecules, and glomerulosclerosis in mice following homocysteine-induced NLRP3 activation (123). Studies have shown that Syk participates in the activation of the Syk/JNK/NLRP3 signaling pathway in high-glucose-induced HK2 cells and rat glomerular mesangial cells, mediating glomerular hypertrophy and mesangial expansion in diabetic rats. Furthermore, Syk can induce apoptosis in HK2 cells. JNK activation translocates into the nucleus, where it alters AP-1 transcription and expression through posttranscriptional mechanisms, potentially leading to insulin resistance (IR), insulin deficiency, hyperglycemia, and a high-glucose-mediated inflammatory cycle, thereby exacerbating the progression of DN. ERK1/2 can also phosphorylate intracellular PLA2, releasing arachidonic acid and eicosanoids, thereby altering renal hemodynamics in DN. Additionally, ERK1/2 can promote mesangial cell proliferation and glomerulonephritis via PKC and PTK, accelerating the progression of DN (124). Literature reports indicate that activation of p38MAPK is essential for NLRP3-mediated IL-1 secretion and plays a critical role in the secretion of IL-1β and IL-18 (125). When activated by the inflammasome, p38MAPK enhances the binding capacity of activator protein-1 (AP-1) and increases TGF-β gene expression, thereby positively regulating p38MAPK signaling through a feedback loop. Consequently, when this pathway is activated and TGF-β is overexpressed, a vicious cycle ensues, promoting mesangial cell proliferation and extracellular matrix accumulation.

##### 4.1.2.2 NLRP3 inflammasome and tubulointerstitial damage in DN

Tubulointerstitial fibrosis is one of the primary causes of DN, with multiple contributing factors, including heavy proteinuria, epithelial-to-mesenchymal transition (EMT) of renal tubular epithelial cells, and interstitial cell infiltration. Wallys Garrido et al. also found that caspase-1, IL-18, IL-6, IL-10, and the pro-fibrotic marker α-SMA were all upregulated, mediating renal injury and proteinuria (126). Kehong Chen et al. discovered in DN renal tubular epithelial cells that the expression of optineurin (OPTN) was negatively correlated with NLRP3 inflammasome activation, which mediated renal interstitial inflammation (127). Overexpression of OPTN promoted mitophagy, thereby inhibiting NLRP3 inflammasome activation. Wenbei Han et al. demonstrated in a rat model of DN that inflammasome activation and TLR4/NF-κB signaling mediated the transdifferentiation of renal tubular epithelial cells (128). Chenxu Ge et al. observed significant insulin resistance and glucose intolerance in an obese animal model, accompanied by renal inflammation and increased expression of IL-1β, IL-18, TNF-α, and IL-6, potentially mediated by NF-κB/NLRP3 signaling, which was further validated in human immortalized renal tubular epithelial cells (129). IncRNA-GM4419 can activate the NF-κB pathway by directly interacting with the p50 subunit of NF-κB, and p50 can also directly interact with the NLRP3 inflammasome (130). Wei Li et al. found that total astragalus extract (TEA) reduced doxorubicin-induced morphological changes, viability loss, and cell death in renal tubular epithelial cells by inhibiting the ROS-ERK1/2-NLRP3 inflammasome axis, strongly indicating that the NLRP3 inflammasome plays a critical role in tubular damage and interstitial fibrosis in DN (131).

##### 4.1.2.3 NLRP3 inflammasome and the treatment of diabetic nephropathy

Current clinical management of diabetic nephropathy (DN) remains anchored in the use of angiotensin-converting enzyme inhibitors (ACEIs) or angiotensin II receptor blockers (ARBs) to inhibit the renin–angiotensin–aldosterone system (RAAS). Although these agents effectively delay disease progression, they are incapable of reversing or eliminating established renal injury (132). Consequently, a rapidly expanding body of research has centered on the NLRP3 inflammasome as a pivotal therapeutic node, seeking to identify interventions that may not only halt but potentially reverse DN by targeting upstream activators and downstream effectors of inflammasome signaling. Targeting the NF-κB signaling pathway: Multiple pharmacological agents have been identified that mitigate renal injury in diabetic nephropathy through suppression of NF-κB signaling. Liquiritigenin alleviates high glucose (HG)-induced extracellular matrix accumulation, oxidative stress, and inflammation by concurrently inhibiting NF-κB and NLRP3 inflammasome pathways (132). The Huangkui capsule reduces tubular epithelial-to-mesenchymal transition (EMT) via blockade of the TLR4/NF-κB signaling axis (128). The insulin-sensitizing agent pioglitazone downregulates the expression of advanced glycation end products (AGEs), their receptor RAGE, and NF-κB, thereby suppressing NLRP3 activation and downstream pro-inflammatory mediators. Fisetin (FIS) inhibits NF-κB activation and ameliorates insulin resistance by targeting receptor-interacting protein kinase 3 (RIP3)-mediated inflammatory signaling (129). Pharmacological blockade of adenosine A3 receptors reduces nuclear translocation of NF-κB and attenuates caspase-1 activation in renal tubular epithelial cells of diabetic rats (126). Thrombomodulin domain 1 (THBD1) protects against DN-associated renal injury by suppressing NF-κB/NLRP3 activation, dampening Nrf2 activity, and reducing podocyte autophagy (133). These findings collectively highlight NF-κB as a master regulatory node whose targeted inhibition may serve as a powerful indirect strategy to suppress NLRP3 inflammasome activation.

Through the inhibition of ROS generation, apocynin, an anti-inflammatory compound, suppresses ROS production and thereby attenuates NLRP3 activation. In DN rat models, apocynin intervention correlates with reduced expression of the X-linked inhibitor of apoptosis protein (XIAP), which parallels decreased NLRP3 levels—suggesting that XIAP may participate in ROS-mediated NLRP3 inflammasome activation (134). The redox-sensitive transcription factor Nrf2 serves as a central endogenous regulator of ROS homeostasis; minocycline and curcumin exert renoprotective effects, at least in part, by modulating Nrf2 activity (101, 135). Multiple herbal extracts, including luteolin (136), curcumin, crocin, cinnamon, and garlic extracts, inhibit NLRP3 inflammasome activation by suppressing ROS generation, mitigating oxidative stress, or enhancing insulin sensitivity (137). Rapamycin activates autophagy, reduces ROS accumulation, and protects podocytes. Optineurin suppresses NLRP3 activation by enhancing mitophagy and reducing mitochondrial ROS (mtROS) production (127). Total extract of astragalus (TEA) inhibits NLRP3 activation by blocking ERK1/2 signaling within the ROS–ERK1/2–NLRP3 axis (57). Current research on ROS inhibitors remains heavily focused on traditional herbal compounds; rigorous mechanistic dissection and comprehensive pharmacotoxicological profiling are essential to accelerating their clinical translation. Targeting NLRP3 inflammasome activity: Minocycline attenuates NLRP3 inflammasome activation by silencing NLRP3 or ASC gene expression or by inhibiting caspase-1 activity. Silencing of TXNIP enhances the expression of antioxidant factors and suppresses high glucose-induced NLRP3 inflammasome activation and podocyte injury (138). Glibenclamide, verapamil, and salidroside inhibit HG-induced TXNIP upregulation and subsequent NLRP3 inflammasome assembly (57, 139). Genetic ablation of NLRP3 reduces the expression of TXNIP and NADPH oxidase 4 (NOX4), enhances superoxide dismutase (SOD) production, and attenuates IL-1β and IL-18 expression (140). NLRP3 silencing (141) further suppresses ROS generation and TGF-β1-induced EMT in renal tubular epithelial cells, restores podocyte autophagy, and ameliorates HG-induced podocyte damage. Li Fang et al. (120) demonstrated that tauroursodeoxycholic acid (TUDCA) enhances endoplasmic reticulum (ER) stress adaptation and reduces NLRP3 activation triggered by proteinuria in DN. MCC950, a highly selective NLRP3 inhibitor, specifically blocks caspase-1-dependent NLRP3 activation and IL-1β secretion without interfering with TLR signaling or the priming phase of inflammasome assembly (142); it improves renal function, reduces mesangial expansion and basement membrane fibrosis, and attenuates tubular dilation—effects achieved independently of changes in body weight or glycemia (143). IL-22 inhibits NLRP3 activation, reduces albuminuria, and attenuates renal fibrosis (144). Genetic deletion of TLR4 mitigates HG-induced podocyte injury and renal damage via suppression of the NLRP3 inflammasome (145). In macrophages, regulated in development and DNA damage response 1 (REDD1), which is partially localized to mitochondria, promotes NLRP3 activation via ROS generation and potentially through NF-κB-dependent mechanisms. Faustine Pasto et al. showed that REDD1 deficiency in macrophages cocultured with adipocytes reduces NLRP3 expression, IL-1β secretion, and insulin resistance (146). Collectively, inhibition of NLRP3 inflammasome activation significantly attenuates renal tissue damage and partially restores renal function; however, clinically viable, tissue-specific NLRP3-targeted therapeutics remain scarce and urgently require further development.

Downstream of inflammasome activation, direct targeting of effector cytokines—particularly IL-1β and IL-18—offers a complementary therapeutic approach. The U.S. Food and Drug Administration (FDA) has approved several IL-1β antagonists, including rilonacept, canakinumab, and anakinra, which reduce glycated hemoglobin levels, enhance insulin secretion, and suppress systemic inflammation in patients with type 2 diabetes, albeit with suboptimal pharmacokinetic profiles (147). Losartan also suppresses IL-1β expression and partially inhibits NLRP3 inflammasome activation (148). Dapagliflozin, an SGLT2 inhibitor, reduces systemic inflammation by lowering circulating levels of C-reactive protein, IL-6, and TNF-α (149). Ginsenoside compound K (CK) inhibits ROS-mediated NLRP3 activation and NF-κB/p38 MAPK signaling and exhibits synergistic effects with MCC950 and VX765 (a caspase-1 inhibitor) in suppressing the IL-1β concentration (150).

With the advancement of research both domestically and internationally, pyroptosis has been firmly established as a critical contributor to the initiation and progression of DKD. Here, we systematically summarize the key molecular components involved in pyroptotic signaling, including inflammasome assembly (notably the NLRP3 inflammasome), activation of the caspase family (particularly caspase-1/4/5/11), and the pore-forming activity of GSDMD. Nevertheless, our current understanding of how pyroptosis mechanistically drives DKD pathogenesis remains incomplete. The precise molecular events governing each step of pyroptotic execution, from inflammasome priming to membrane rupture, have not yet been fully elucidated. Moreover, the functional significance of pyroptosis-induced cell death in the context of DKD progression is still largely confined to preclinical models. Therefore, comprehensive and mechanistic investigations are urgently needed to delineate the specific roles and regulatory networks of pyroptosis and inflammasome activation in DKD—insights that may ultimately reveal novel therapeutic targets for this devastating complication of diabetes.

#### 4.1.3 The role and mechanisms of the NLRP3 inflammasome in IgA nephropathy

Immunoglobulin A (IgA) nephropathy is currently recognized as the most prevalent primary glomerular disease worldwide, with 20%–40% of patients progressing to end-stage kidney disease within 20 years of diagnosis (151). The pathogenesis is primarily attributed to the mesangial deposition of immune complexes containing aberrantly glycosylated IgA1, followed by T lymphocyte-mediated inflammatory responses; innate immune mechanisms also contribute significantly to disease initiation and progression (152). Nucleotide-binding oligomerization domain-like receptor protein 3 (NLRP3), a recently identified pattern recognition receptor, is expressed in multiple resident renal cells—including tubular epithelial cells, mesangial cells, and podocytes. Upon activation by exogenous or endogenous stimuli, NLRP3 assembles with the adaptor protein apoptosis-associated speck-like protein containing a CARD (ASC) to form the NLRP3 inflammasome. This complex activates caspase-1, which in turn drives the maturation and secretion of the pro-inflammatory cytokines interleukin-18 (IL-18) and interleukin-1β (IL-1β), thereby amplifying local and systemic inflammation (153, 154). A growing body of evidence indicates that the NLRP3 inflammasome plays a pivotal role in both the initiation and progression of IgA nephropathy and is intimately linked to injury of intrinsic renal cells—including podocytes, mesangial cells, glomerular endothelial cells, and tubular epithelial cells (155–157). Notably, certain traditional Chinese herbal medicines exert renoprotective effects in IgA nephropathy by targeting the NLRP3 inflammasome and its downstream signaling components, thereby modulating inflammatory cytokine production and associated pathways to attenuate disease progression.

##### 4.1.3.1 The role of the NLRP3 inflammasome in IgA nephropathy

An expanding body of evidence demonstrates that the NLRP3 inflammasome contributes to the pathogenesis of multiple kidney diseases, including IgA nephropathy (154, 158). In patients with IgA nephropathy, circulating levels of NLRP3 inflammasome-derived cytokines, notably interleukin-18 (IL-18) and interleukin-1β (IL-1β), are significantly elevated (154, 159), underscoring the inflammasome’s central role in disease progression. Targeted inhibition of NLRP3 within the kidney has therefore emerged as a promising therapeutic strategy for IgA nephropathy (156). Further mechanistic insights reveal that colorectal neoplasia differentially expressed (CRNDE), a long non-coding RNA, exacerbates IgA nephropathy by promoting NLRP3 inflammasome activation in macrophages; conversely, CRNDE suppression enhances NLRP3 degradation, thereby attenuating renal inflammation (155). Clinically, peripheral blood mononuclear cells from IgA nephropathy patients exhibit elevated NLRP3 mRNA expression, which correlates positively with renal fibrosis indices (160). Moreover, serum exosomes from these patients show markedly increased NLRP3 levels, which correlate positively with proteinuria severity and Katafuchi histological scores and negatively with the estimated glomerular filtration rate (eGFR). Importantly, NLRP3 inflammasome expression within renal tissue is significantly upregulated and strongly correlates with its levels in circulating exosomes (161). Within the tubulointerstitium of IgA nephropathy kidneys, the expression of the NLRP3 inflammasome, IL-18, and monocyte chemoattractant protein-1 (MCP-1) is markedly increased and positively correlates with the degree of proteinuria, tubular atrophy, interstitial inflammatory cell infiltration, and fibrosis (162). The activation of the NLRP3 inflammasome in IgA nephropathy is orchestrated through multiple interconnected pathways—including NF-κB signaling, impaired autophagy, mitochondrial reactive oxygen species (mtROS) overproduction, and exosome-mediated intercellular communication (154). Critically, injury or dysfunction of intrinsic renal cells, including podocytes, mesangial cells, glomerular endothelial cells, and tubular epithelial cells, is closely linked to activation of the NLRP3 inflammasome.

##### 4.1.3.2 NLRP3 and podocyte injury

Podocytes constitute the final filtration barrier of the glomerulus. Their injury—manifested ultrastructurally by foot process effacement and detachment, and histologically by hypertrophy, focal sclerosis, Bowman’s capsule adhesion, and podocyte loss—represents a hallmark lesion in IgA nephropathy and a key driver of proteinuria and progressive renal decline (163–165). Podocyte injury is now widely recognized as a central mechanism underlying disease progression in IgA nephropathy (163, 164). Emerging evidence indicates that IgA1-containing immune complexes directly trigger NLRP3 inflammasome activation in both macrophages and podocytes in IgA nephropathy (159). Compared with healthy controls, renal tissue from IgA nephropathy patients exhibits significantly elevated NLRP3 inflammasome expression. Notably, co-localization of NLRP3 with the macrophage marker F4/80 is detectable within podocytes, suggesting phenotypic transition. Patients with an estimated glomerular filtration rate (eGFR) < 60 mL·min^−1^·(1.73 m²)^−1^ show markedly increased tubular NLRP3 expression, whereas those with heavy proteinuria (≥3.5 g·day^−1^) exhibit significantly elevated glomerular NLRP3 levels. Critically, aberrantly glycosylated IgA1 isolated from the serum of IgA nephropathy patients induces NLRP3 expression in cultured podocytes and upregulates F4/80—a macrophage lineage marker—concomitant with increased expression of the adhesion molecule vascular cell adhesion molecule-1 (VCAM-1) and the fibrotic marker α-smooth muscle actin (α-SMA). These findings indicate that pathogenic IgA1 not only activates the NLRP3 inflammasome in podocytes but also initiates podocyte-to-macrophage transdifferentiation (PMT). Following PMT, these transformed podocytes secrete pro-inflammatory cytokines that amplify inflammatory cascades and promote renal fibrosis—key pathological features of IgA nephropathy (158).

##### 4.1.3.3 NLRP3 and mesangial cell proliferation

Mesangial cells reside between glomerular capillaries, embedded within the mesangial matrix. They maintain direct contact with endothelial cells and intimate crosstalk with podocytes, collectively forming the functional architecture of the glomerulus. Disruption of mesangial cell homeostasis, whether by immune complexes, hemodynamic stress, or metabolic insults, triggers their pathological activation. This activation drives mesangial cell proliferation and hypertrophy, expansion of the extracellular matrix, release of pro-inflammatory mediators, and complement activation, ultimately culminating in mesangiolysis and loss of glomerular capillary loops, thereby impairing glomerular filtration (166). In IgA nephropathy, aberrant deposition of IgA within the mesangium serves as a potent trigger for NLRP3 inflammasome activation, initiating a cascade of localized inflammation that promotes mesangial cell hyperproliferation and excessive extracellular matrix accumulation—key histopathological features driving progressive glomerular injury. This IgA–NLRP3 axis is now widely regarded as a central pathogenic mechanism in IgA nephropathy. Tripartite motif (TRIM) proteins, a family of E3 ubiquitin ligases, play critical regulatory roles in innate immunity. Using an *in vitro* model of human glomerular mesangial cells (GMCs) stimulated with pathogenic IgA1, researchers demonstrated that IgA1 promotes GMC proliferation via NLRP3 inflammasome activation. Notably, TRIM40 suppresses IgA1-induced GMC proliferation by inhibiting NLRP3 inflammasome assembly and downstream signaling (167). Furthermore, in a cellular model of IgA nephropathy established by culturing human renal tubular epithelial cells (HK-2 cells) with conditioned medium from IgA-stimulated human mesangial cells (HMCs), NLRP3 mRNA and protein expressions were significantly upregulated in HK-2 cells, accompanied by increased levels of ASC and caspase-1-indicating that mesangial-derived inflammatory signals can propagate NLRP3 activation to tubular compartments, thereby linking glomerular injury to tubulointerstitial inflammation (168).

##### 4.1.3.4 NLRP3 and glomerular endothelial cell injury

Clinical studies consistently report that endothelial damage, often accompanied by endothelial cell loss, is a hallmark histopathological feature of IgA nephropathy (169). In acute glomerular lesions of IgA nephropathy, endothelial cell proliferation, fibrinoid necrosis, and the presence of cellular or fibrocellular crescents are strongly associated with hematuria, with or without concurrent proteinuria. In chronic lesions, segmental or global glomerulosclerosis correlates significantly with the severity of proteinuria and elevated serum creatinine levels. Collectively, injury to glomerular capillaries and loss of endothelial integrity in both acute and chronic phases of IgA nephropathy are thought to directly contribute to hematuria, proteinuria, and progressive renal dysfunction (169). In animal models of IgA nephropathy, ultrastructural abnormalities such as endothelial vacuolization and mesangial interposition have been observed, further supporting the role of endothelial injury in disease progression (170). Galactose-deficient IgA1 (Gd-IgA1) immune complexes exhibit high affinity for glomerular endothelial cells. Their deposition triggers glycocalyx shedding and disrupts the glomerular filtration barrier. Moreover, Gd-IgA1 complexes accelerate the production of adhesion molecules and pro-inflammatory cytokines in endothelial cells. This endothelial damage, induced by Gd-IgA1 deposition, may enhance the permeability of mesangial regions to immunoglobulins and amplify subsequent inflammatory responses—thereby potentiating core pathogenic mechanisms in IgA nephropathy (171). *In vitro* models using human glomerular endothelial cells exposed to high glucose demonstrate robust activation of the NLRP3 inflammasome, accompanied by excessive secretion of IL-18 and IL-1β—suggesting that metabolic stress synergizes with immune injury to exacerbate endothelial dysfunction via inflammasome signaling (172). Notably, retinoic acid receptor responder 1 (Rarres1) is detectably expressed in glomerular and peritubular capillary endothelial cells in IgA nephropathy and related glomerulopathies. Induction of Rarres1 in endothelial cells represents a conserved molecular mechanism that drives inflammation and fibrosis through activation of the NF-κB signaling pathway (173).

##### 4.1.3.5 NLRP3 and tubular epithelial cell injury

In IgA nephropathy, injury to renal tubular epithelial cells primarily arises from glomerular filtration barrier dysfunction and pathological crosstalk between mesangial and tubular compartments. Filtered proteins, including albumin (ALB), complement components, cytokines, growth factors, and galactose-deficient IgA1 (Gd-IgA1), play pivotal roles in driving tubulointerstitial damage. These filtered molecules stimulate proximal tubular epithelial cells to secrete a spectrum of inflammatory mediators, thereby establishing a pro-inflammatory microenvironment within the tubulointerstitium (174). Crosstalk between mesangial cells and tubular epithelial cells is mediated by key signaling molecules, including TNF-α, TGF-β1, and MCP-1 (175). NLRP3 is expressed in human kidney biopsy specimens and in primary human proximal tubular cells (HPTCs), and its expression levels correlate with clinical outcomes in IgA nephropathy. In healthy human kidneys, NLRP3 is predominantly localized to renal tubules and, within human proximal tubular cells (HPTCs), to mitochondria. Compared with control kidneys, renal tissues from patients with IgA nephropathy exhibit significantly elevated NLRP3 gene expression. Although NLRP3 protein can be detected in glomeruli, its expression is primarily confined to the tubular epithelial compartment. *In vitro*, stimulation of HPTCs with TGF-β1 transiently induces NLRP3 mRNA and protein expression. However, over time, these cells undergo phenotypic transition, losing their epithelial identity through transcriptional reprogramming and ubiquitin-mediated degradation, which coincides with progressive downregulation of NLRP3 expression. Consistent with these *in vitro* findings, low NLRP3 mRNA expression in renal biopsies correlates with a linearly increased risk of the composite endpoint of serum creatinine doubling and progression to end-stage kidney disease in IgA nephropathy patients (176). Collectively, these data indicate that NLRP3 is predominantly a tubule-expressed protein in the human kidney, and its expression is paradoxically reduced in progressive IgA nephropathy.

##### 4.1.3.6 Therapeutic modulation of the NLRP3 inflammasome in IgA nephropathy by natural compounds

Tripterygium wilfordii (Lei Gong Teng) is widely used in the treatment of inflammatory and autoimmune diseases. Extensive clinical, animal, and *in vitro* studies confirm its potent anti-inflammatory effects (177, 178). Mechanistically, Tripterygium and its bioactive constituents modulate immune cell function and suppress expression of cytokines, adhesion molecules, and inflammatory mediators through multiple signaling pathways—including NF-κB, MAPK, STAT, NLRP3 inflammasome, and Wnt (179). Diterpenoids 1 and 6 isolated from Tripterygium inhibit LPS-induced inflammation in murine macrophages by suppressing MAPK and NF-κB signaling and STAT3 activation, thereby reducing NLRP3 inflammasome assembly and expression of inflammatory mediators such as COX-2, iNOS, IL-6, IL-1β, and IL-18 (180). Triptolide, a principal bioactive diterpenoid epoxide from Tripterygium, exhibits the strongest anti-inflammatory and immunosuppressive activity among its constituents (181). In IgA nephropathy rat models, triptolide significantly reduces serum creatinine (SCr), blood urea nitrogen (BUN), and 24-h urinary protein excretion. It also lowers serum levels of TNF-α, IL-17A, interferon-γ (IFN-γ), and IL-4, attenuates renal IgA deposition, and suppresses renal expression of IL-1β, caspase-1, IL-18, and NLRP3—suggesting its renoprotective effects are mediated, at least in part, through inhibition of NLRP3 inflammasome activation (182). Triptolide’s anti-inflammatory action is further linked to suppression of the NLRP3/TLR4 axis, reducing IL-1β and IL-18 levels, limiting immune complex deposition and mesangial proliferation, and ameliorating proteinuria (183). Celastrol, a quinone methide triterpenoid extracted from Tripterygium root bark, possesses anti-inflammatory, immunosuppressive, and antitumor activities (184). It inhibits NF-κB signaling, downregulates NLRP3 expression, and blocks caspase-1 cleavage, thereby suppressing IL-1β and IL-18 production in LPS-stimulated macrophages (185). In IgA nephropathy models, celastrol attenuates hematuria and proteinuria by inhibiting the Notch signaling pathway in renal tissue (186). Wogonoside alleviates mesangial cell proliferation and matrix expansion in IgA nephropathy rats. It elevates cytoplasmic NF-κB levels while reducing nuclear NF-κB translocation and dose-dependently lowers SCr, BUN, IL-1β, TNF-α, 24-h urinary protein, and red blood cell counts. It also suppresses the renal expression of nuclear NF-κB, nuclear/total NF-κB ratio, NLRP3, ASC, pro-caspase-1, and caspase-1 (187). Baicalin reduces BUN, SCr, and 24-h urinary protein in rats with mesangial proliferative glomerulonephritis. It decreases the kidney-to-body weight ratio, glomerular apoptosis rate, and renal mRNA and protein levels of NLRP3 and caspase-1 (43, 188). Plumbagin significantly reduces urinary protein, SCr, and BUN in IgA nephropathy rats. It attenuates renal oxidative stress by lowering ROS and malondialdehyde (MDA) levels while enhancing superoxide dismutase (SOD) activity. Plumbagin also reduces serum MDA, IL-1β, IL-18, and TNF-α and downregulates renal expression of NLRP3, ASC, caspase-1, PI3K, Akt, and NF-κB (49, 189). In a separate study, plumbagin suppressed apoptosis and oxidative stress in renal tissue, reduced pro-IL-1β and pro-IL-18 levels, and inhibited NLRP3/ASC/caspase-1 protein expression (190). It also inhibits proliferation of human mesangial cells and downregulates the expression of TGF-β1, CTGF, and fibronectin (FN) (191). Geniposide dose-dependently reduces 24-h urinary protein, BUN, and SCr in IgA nephropathy mice. It attenuates IgA deposition, mesangial expansion, and inflammatory cell infiltration, while suppressing renal oxidative stress and inflammation. Geniposide significantly reduces renal NLRP3 protein expression. Notably, NLRP3 knockout (KO) mice exhibit similar protective effects as geniposide treatment (100 mg/kg), whereas geniposide shows no additional benefit in NLRP3 KO mice—strongly implicating NLRP3 as its primary molecular target (192). Icariin, a flavonoid from epimedium, reduces urinary red blood cells, proteinuria, and urinary N-acetyl-β-D-glucosaminidase (NAG) in experimental IgA nephropathy rats. It diminishes renal IgA deposition and suppresses renal protein expression of NF-κB p65 and MCP-1, as well as mRNA levels of IL-4, IL-10, and IL-13 (193). Icariin also lowers serum IL-1β, IL-6, and IL-18, reduces renal expression of TGF-β1, collagen IV (Col IV), and FN1, and inhibits nuclear translocation of NF-κB p65, TNF-α, and VCAM-1 (194). Its renoprotective mechanism involves blockade of NF-κB nuclear translocation and NLRP3 inflammasome activation, thereby reducing downstream pro-inflammatory cytokine production (195). Artemisinin, derived from Artemisia annua, alleviates renal injury in IgA nephropathy mice. Network pharmacology and molecular docking analyses, validated experimentally, suggest that artemisinin activates the Akt/Nrf2 signaling pathway to exert therapeutic effects (196). Artemisinin significantly reduces 24-h urinary protein and hematuria, lowers serum creatinine, BUN, total cholesterol, and triglycerides, while increasing serum albumin and total protein. It suppresses renal production of IL-4 and IL-17, ameliorates glomerular mesangial matrix expansion and cell proliferation, and protects renal structure. Mechanistically, artemisinin enhances exosome secretion, which in turn inhibits NF-κB/NLRP3 inflammasome activation (197). When combined with hydroxychloroquine, artemisinin further amplifies exosome release from tubular epithelial cells; upon uptake by mesangial cells, these exosomes suppress NF-κB signaling and NLRP3 inflammasome activity, downregulating the expression of IκBα, p-p65, NLRP3, ASC, IL-1β, and caspase-1, ultimately attenuating renal inflammation (197). Emerging evidence indicates that ROS generation, coupled with activation of NF-κB and the NLRP3 inflammasome, constitutes a central pathogenic axis driving the progression of IgA nephropathy (198). In murine models of IgA nephropathy, treatment with osthole, a bioactive coumarin derivative, confers significant renoprotection: It prevents proteinuria, improves renal function, and halts progressive histopathological lesions, including glomerular hypercellularity, glomerulosclerosis, and periglomerular monocyte infiltration. Mechanistically, osthole reduces renal superoxide anion levels and promotes nuclear translocation of the antioxidant transcription factor Nrf2. It concurrently suppresses activation of NF-κB and the NLRP3 inflammasome in renal tissue, leading to decreased expression of MCP-1 and reduced monocyte infiltration. *In vitro*, osthole inhibits ROS production and NLRP3 inflammasome activation in stimulated macrophages. In activated mesangial cells, it similarly attenuates ROS generation and downregulates MCP-1 protein expression. Collectively, these findings demonstrate that osthole exerts its therapeutic effects in IgA nephropathy primarily by targeting renal oxidative stress and interrupting the ROS–NF-κB–NLRP3 inflammatory cascade—positioning it as a promising multi-target natural agent for disease modification.

#### 4.1.4 Role and mechanisms of the NLRP3 inflammasome in lupus nephritis

Systemic lupus erythematosus (SLE) is a chronic autoimmune disease characterized by the production of pathogenic autoantibodies, lymphocyte hyperproliferation, and inflammatory injury to multiple organs. Renal involvement, manifesting as LN, is a frequent and severe complication, presenting clinically with proteinuria, hematuria, progressive renal dysfunction, and, in advanced cases, end-stage kidney failure. LN remains a leading cause of mortality in SLE patients (199). The pathogenesis of LN is highly complex, involving both innate and adaptive immune-mediated inflammatory cascades that converge on renal tissue destruction. Central to this process is the activation of the NLRP3 inflammasome, which amplifies local and systemic inflammation through multiple interconnected pathways. In SLE, immune complexes containing anti-DNA or anti-RNA autoantibodies activate the NLRP3 inflammasome in monocytes and macrophages via two synergistic mechanisms: (i) upregulation of TLR-dependent NF-κB signaling, which primes inflammasome component expression, and (ii) induction of mtROS, which provides the critical second signal for NLRP3 assembly. This dual activation drives enhanced secretion of IL-1β and IL-17, thereby exacerbating systemic autoimmunity and renal inflammation (200). Additionally, complement component C3a, generated upon immune complex-mediated complement activation, stimulates ATP release from macrophages and dendritic cells. Extracellular ATP then engages the purinergic receptor P2X7, triggering NLRP3 inflammasome assembly and subsequent IL-1β maturation and secretion. This pathway further amplifies inflammatory injury in LN and contributes to disease progression (200, 201).

##### 4.1.4.1 NLRP3 inflammasome in the pathogenesis of lupus nephritis

Under physiological conditions, inflammatory responses serve to eliminate pathogens and promote tissue repair. However, dysregulated or excessive inflammation can inflict significant tissue damage. Although the NLRP3 inflammasome, a key component of the innate immune system, plays a critical role in host defense against infection, its hyperactivation contributes to the pathogenesis of multiple autoimmune diseases, including LN. Clinical evidence demonstrates that the expression levels of NLRP3 and caspase-1 are significantly elevated in renal biopsies from LN patients (202). Moreover, NLRP3 mRNA levels are markedly upregulated in LN kidney tissue and inversely correlate with renal function (71). These clinical observations are further supported by robust experimental data from murine lupus models. In (NZB×NZW)F1 lupus-prone mice, renal NLRP3 inflammasome activation is markedly enhanced (203). Zhao et al. confirmed pronounced upregulation of the NLRP3 inflammasome in the kidneys of MRL/lpr mice; notably, pharmacological or genetic inhibition of NLRP3 attenuates disease severity in this model (204). Conversely, forced overexpression of NLRP3 exacerbates end-organ damage in lupus mice, underscoring its pathogenic role in LN progression (205). Earlier work from our group demonstrated that pharmacological inhibition of pro-caspase-1 activation reduces IL-18 production, subsequently dampening IFN-γ secretion, and confers significant protection in murine LN models (206). Kahlenberg et al. were the first to employ genetic knockout models to dissect inflammasome function in lupus. Compared with wild-type mice, caspase-1^−^/^−^ lupus mice exhibit significantly reduced serum titers of anti-dsDNA antibodies and anti-ribonucleoprotein antibodies, along with attenuated type I interferon responses, thereby decreasing immune complex formation and subsequent renal damage (207). This suggests that caspase-1 is involved in the pathogenesis of LN. The pathogenic roles of NLRP3-derived cytokines IL-1β and IL-18 in SLE have long been established in preclinical models (208–210). Our earlier studies further revealed that plasma and renal IL-18 levels positively correlate with proteinuria, histopathological damage, and IgG immune complex deposition in BXSB lupus mice—suggesting that elevated IL-18 may directly contribute to glomerular filtration barrier disruption and autoimmune renal injury in LN (211). Interleukin-18 binding protein (IL-18BP), the endogenous high-affinity antagonist of IL-18, is significantly upregulated in both renal tissue and peripheral blood of LN patients. Importantly, an imbalance between IL-18 and IL-18BP may actively contribute to lupus pathogenesis (212, 213). Recent clinical studies corroborate these findings, reporting significantly elevated serum levels of IL-1β and IL-18 in SLE patients—highlighting the clinical relevance of inflammasome-derived cytokines in disease progression (214, 215). Intriguingly, loss of NLRP3 function has also been linked to autoimmune dysregulation. Sester et al. serendipitously discovered a spontaneous NLRP3 mutation in NZB mice (216). This mutation may alter host–microbiome interactions and promote the generation of autoreactive antibodies. More directly, Lech et al. demonstrated that genetic deletion of NLRP3 or ASC in lupus-prone mice leads to hyperactivation of dendritic cells and macrophages, excessive production of pro-inflammatory mediators, and accelerated T- and B-cell proliferation (217). Mechanistically, NLRP3 and ASC deficiency strongly suppresses TGF-β receptor-mediated immunosuppressive signaling. Collectively, these findings reveal a paradoxical duality of the NLRP3 inflammasome in autoimmunity: Basal, homeostatic NLRP3 activity may be essential for immune tolerance and suppression of aberrant lymphocyte activation; however, sustained or excessive NLRP3 inflammasome activation drives cytokine storm, immune dysregulation, and target organ damage—thereby fueling LN progression. This delicate balance must be carefully considered in the development of NLRP3-targeted therapies for LN: Complete inhibition may risk unleashing compensatory hyperinflammation, whereas selective or context-dependent modulation may offer optimal therapeutic benefit.

##### 4.1.4.2 Renal injury mechanisms mediated by the NLRP3 inflammasome in lupus nephritis

The evidence outlined above establishes that core components of the NLRP3 inflammasome—including NLRP3, ASC, and caspase-1—are critically involved in the pathogenesis of LN. Below, we delineate how activated NLRP3 inflammasome signaling drives renal injury by modulating both circulating immune cells and intrinsic renal cells. A central mechanism involves the NLRP3 inflammasome’s regulation of CD4^+^ T-cell differentiation—particularly the Th1 and Th17 subsets, which are key drivers of renal inflammation in LN. The inflammasome promotes Th1 and Th17 polarization primarily through caspase-1–dependent secretion of IL-1β and IL-18. In both human and murine systems, IL-1β—in synergy with TGF-β—induced the expression of the transcription factors IRF4 and RORγt, thereby promoting the differentiation of naïve CD4 T cells into pathogenic Th17 cells. Conversely, IL-18 acting in concert with IL-12 drives naïve CD4 T cells toward a Th1 fate characterized by IFN-γ production. Notably, IL-18 itself is also secreted by Th1 cells, creating a self-amplifying inflammatory loop. In experimental autoimmune encephalomyelitis (EAE), a Th1/Th17-driven model, genetic ablation of NLRP3 significantly attenuates disease severity by dampening Th1- and Th17-mediated immune responses. Further mechanistic insight comes from studies of ASC-deficient CD4 T cells, which paradoxically secrete elevated levels of the immunoregulatory cytokine IL-10. This IL-10 surge suppresses proliferation of neighboring T cells and inhibits their production of IFN-γ and IL-2, highlighting a cell-intrinsic immunosuppressive function of ASC in T cells (218).

Activation of the NLRP3 inflammasome also critically contributes to the pathogenesis of LN by modulating the function of macrophages and dendritic cells. Infiltrating renal macrophages exacerbate glomerular injury and tubulointerstitial inflammation through the secretion of IL-1β and IL-18. In SLE, abundant neutrophil extracellular traps (NETs) are released into the circulation. These NETs directly activate the NLRP3 inflammasome in both human and murine macrophages, triggering robust IL-1β and IL-18 secretion. In turn, IL-18 stimulates neutrophils to generate additional NETs—establishing a self-amplifying positive feedback loop that accelerates systemic inflammation in SLE (216). During SLE flares, cell death releases double-stranded DNA (dsDNA), which, together with subsequently generated anti-dsDNA autoantibodies, engages TLR4 on monocytes and macrophages. This interaction induces ROS production and potassium efflux, both of which serve as canonical triggers for NLRP3 inflammasome assembly. Notably, pharmacological antioxidants or genetic downregulation of TLR4 significantly suppresses NLRP3 activation in these myeloid cells (219). More recently, it has been demonstrated that reduced serum high-density lipoprotein (HDL) levels in SLE patients impair cholesterol efflux in dendritic cells. This lipid dysregulation activates the NLRP3 inflammasome, enhancing secretion of pro-inflammatory cytokines and promoting polarization of CD4^+^ T cells toward Th1 and Th17 phenotypes. Crucially, knockdown of NLRP3 expression in dendritic cells markedly attenuates these inflammatory and immunomodulatory effects, underscoring the central role of NLRP3 in bridging lipid metabolism, innate immunity, and adaptive T-cell responses in SLE (220).

##### 4.1.4.3 NLRP3 inflammasome activation in intrinsic renal cells drives lupus nephritis

It has been firmly established through both *in vivo* and *in vitro* studies that intrinsic renal cells in humans and rodents, including podocytes (221–223), mesangial cells (121, 224), glomerular endothelial cells (68), and tubular epithelial cells (225, 226), are capable of expressing the NLRP3 inflammasome. Upon activation, these cells cleave pro-caspase-1 into its active form, leading to the maturation and secretion of pro-inflammatory cytokines such as IL-1β and IL-18—thereby directly contributing to local renal inflammation and tissue injury. Below, we focus on the impact of NLRP3 inflammasome activation in tubular epithelial cells and podocytes, two key cellular compartments in LN. Tubulointerstitial inflammation is a critical determinant of disease progression in LN. Upon injury, damaged or necrotic tubular epithelial cells release DAMPs—including ROS, extracellular ATP, uric acid, nucleic acids, and extracellular matrix components (e.g., hyaluronan, biglycan). These DAMPs activate the NLRP3 inflammasome within neighboring tubular epithelial cells, triggering the release of inflammatory cytokines and chemokines that recruit neutrophils, macrophages, natural killer cells, and lymphocytes into the renal interstitium. This cascade amplifies local inflammation and accelerates tubulointerstitial injury (227). Our previous work demonstrated that elevated IL-18 expression in tubular epithelial cells correlates strongly with the severity of tubulointerstitial damage in LN (228). Faust et al. further confirmed that upregulation of IL-18 in renal tubules positively correlates with histological and functional kidney injury in murine LN models (229, 230). In LN, anti-dsDNA autoantibodies can bind directly to tubular epithelial cells, promoting tubulointerstitial inflammation, a process likely mediated, at least in part, by NLRP3 inflammasome activation. Moreover, NLRP3 signaling in tubular cells not only drives inflammation but also contributes to tissue repair and, paradoxically, to maladaptive fibrogenesis (231). Thus, the NLRP3 inflammasome in tubular epithelial cells is intimately linked to both the inflammatory and fibrotic phases of tubulointerstitial injury in LN. Podocytes are essential for maintaining the structural and functional integrity of the glomerular filtration barrier—and are consistently targeted in LN. Zhang et al. first demonstrated in a hyperhomocysteinemia model that NLRP3 inflammasome activation in podocytes induces foot process effacement, contributing to glomerulosclerosis and proteinuria (221). Shahzad et al. further showed that NLRP3 activation in glomerular intrinsic cells (particularly podocytes) exacerbates glomerular injury in murine models of diabetic nephropathy, highlighting a conserved pathogenic role across glomerulopathies (231). In the context of SLE and LN, recent groundbreaking work from Prof. Niansheng Yang’s team revealed that podocyte NLRP3 is activated by ROS in both LN patients and murine models. Critically, this activation directly mediates podocyte injury and contributes to disease pathogenesis—underscoring the indispensable role of podocyte-intrinsic NLRP3 signaling in LN progression (67).

##### 4.1.4.4 Therapeutic targeting of the NLRP3 inflammasome in lupus nephritis: emerging opportunities

As outlined above, the NLRP3 inflammasome plays a pivotal role in the pathogenesis of LN, offering novel avenues for targeted therapeutic intervention. Numerous studies have explored pharmacological agents capable of suppressing NLRP3 inflammasome activation, including inhibitors of its assembly and regulators of IL-1β and IL-18 secretion; however, most of these agents lack specificity for the NLRP3 pathway. Recently, however, two highly selective inhibitors targeting the NLRP3–ASC–caspase-1–IL-1β/IL-18 axis have emerged, holding significant promise for treating NLRP3-driven diseases, including LN and other autoimmune conditions. β-Hydroxybutyrate (BHB), a ketone body produced during fasting or caloric restriction, directly inhibits NLRP3 inflammasome activation by blocking potassium efflux and ASC oligomerization. Notably, BHB’s inhibitory effect is independent of its chirality and does not rely on classical starvation-associated pathways—including AMPK signaling, ROS modulation, autophagy, or glycolysis inhibition. In both murine and human macrophage models, BHB significantly reduces LPS-induced secretion of IL-1β and other inflammatory cytokines. In NLRP3-dependent murine disease models, including Muckle–Wells syndrome, familial cold autoinflammatory syndrome, and monosodium urate crystal-induced peritonitis, BHB consistently suppresses caspase-1 activation and IL-1β release, confirming its broad anti-inflammatory efficacy (232). MCC950 is a small-molecule inhibitor that selectively blocks both canonical and non-canonical NLRP3 inflammasome activation, without affecting AIM2, NLRC4, or NLRP1 inflammasomes (142). In preclinical models, MCC950 reduces systemic IL-1β levels and ameliorates disease severity in experimental autoimmune encephalomyelitis. Critically, in the NZM2328 murine model of LN, MCC950 treatment significantly attenuates podocyte foot process effacement, improves renal histopathology, and reduces proteinuria—providing direct evidence of its renoprotective potential in lupus nephritis (47). Accumulating evidence underscores the indispensable role of the NLRP3 inflammasome in LN pathogenesis. DAMPs generated during immune dysregulation activate NLRP3 in both circulating immune cells and intrinsic renal cells, leading to caspase-1–dependent maturation and secretion of IL-1β and IL-18. Despite extensive research, the precise molecular mechanisms by which NLRP3 contributes to LN progression remain incompletely defined, necessitating further mechanistic and clinical investigations. While the development of pathway-specific inhibitors, such as BHB and MCC950, offers exciting therapeutic potential, their efficacy and safety in human LN patients remain to be rigorously validated in clinical trials. Translating these preclinical successes into clinical reality will require sustained, multidisciplinary efforts. In summary, as our understanding of the NLRP3 inflammasome in LN continues to deepen, it will pave the way for novel, mechanism-based therapies, not only for LN but also for a broad spectrum of NLRP3-driven autoimmune and inflammatory diseases.

### 4.2 The inflammasome in AKI

The NLRP3 inflammasome plays a critical and context-dependent role in the pathogenesis of AKI. In murine models of ischemia–reperfusion injury (IRI), renal tubular epithelial cells exhibit markedly increased NLRP3 expression, accompanied by histopathological features of tubular necrosis, brush border loss, and tubular dilation. These changes correlate with significant elevations in serum creatinine, blood urea nitrogen (BUN), and urinary protein excretion, collectively indicating that NLRP3 inflammasome activation in tubular epithelial cells is a key driver of ischemia–reperfusion injury (IRI)-induced renal damage (58). The NLRP3 inflammasome also contributes to AKI triggered by contrast agents and rhabdomyolysis. Genetic silencing of NLRP3 significantly ameliorates tubular epithelial cell degeneration, apoptosis, and inflammatory cell infiltration in these models (233, 234). Cao et al. demonstrated that in murine models of sepsis-induced AKI, renal expression of NLRP3, ASC, and caspase-1 is markedly upregulated, accompanied by robust neutrophil infiltration and elevated serum creatinine. Notably, NLRP3 knockout reverses neutrophil accumulation and attenuates creatinine elevation, highlighting its pathogenic role in septic AKI (235). Consistent with preclinical findings, elevated NLRP3 expression has been detected in renal biopsy specimens from patients with crescentic glomerulonephritis and acute tubular necrosis (236). Interestingly, however, the IRI model established by Iyer et al. revealed that NLRP3 knockout significantly ameliorated renal function injury and reduced neutrophil infiltration in the renal interstitium, whereas ASC knockout mice showed no significant effect on these AKI-related changes, suggesting that the NLRP3 inflammasome may exert additional functions independent of ASC and caspase-1 (237). The study by Kim et al. demonstrated that NLRP3 knockout mice were resistant to ischemia–reperfusion injury (IRI)-induced AKI; however, in cisplatin-induced AKI, NLRP3 deficiency did not reduce caspase-1 levels. In fact, caspase-1 activity increased, and tubular necrosis, tubular cell apoptosis, blood urea nitrogen, and serum creatinine levels were not significantly attenuated (238). This indicates that NLRP3 may play a relatively minor role in cisplatin-induced AKI. In contrast, NLRP1 and ASC were upregulated in cisplatin-induced AKI, suggesting that NLRP1 may be functionally involved in this form of AKI. Thus, NLRP3 appears to exert distinct roles in AKI depending on the underlying etiology.

### 4.3 The NLRP3 inflammasome participates in renal ischemia–reperfusion injury via associated inflammatory signaling pathways

Renal ischemia–reperfusion injury (IRI), commonly encountered during kidney transplantation, represents a critical pathophysiological process leading to acute kidney failure and significantly impairs recipient prognosis (239). Inflammatory responses play a pivotal role in the pathogenesis and progression of IRI. The activated NLRP3 inflammasome modulates systemic inflammatory responses and associated cellular functions by mediating the maturation and release of multiple pro-inflammatory cytokines (240). Kidney transplantation remains the optimal therapeutic strategy for end-stage renal disease, with transplant recipients exhibiting markedly superior long-term survival and quality of life compared with patients undergoing dialysis. Nevertheless, renal IRI frequently accompanies kidney transplantation procedures and severely compromises recipient outcomes. IRI is a pathological state in which temporary reduction of blood supply to an organ, followed by restoration of perfusion, paradoxically induces functional impairment and even structural damage (241). The kidney is among the primary organs vulnerable to IRI, with underlying mechanisms potentially involving inflammatory responses, oxygen free radicals, intracellular calcium overload, apoptosis, and other factors (242). Following renal ischemia, dysfunction of tubular epithelial cells and endothelial cells activates leukocytes within the tissue, ultimately exacerbating vascular leakage and interstitial edema (243). Upon subsequent stimulation, the host can activate pattern recognition receptors such as Toll-like receptors (TLRs) and NOD-like receptors (NLRs) to exert inflammatory regulatory effects (244). NLRs constitute a family of intracellular innate sensors—cytoplasmic proteins that regulate inflammatory and apoptotic responses. Among them, the NLRP3 inflammasome has attracted particular research attention. The NLRP3 inflammasome contributes to the progression of renal IRI by amplifying inflammatory responses in immune cells and modulating interactions between immune cells and non-immune renal cells (245). Elucidating the inflammatory signaling pathways associated with the NLRP3 inflammasome in renal IRI may hold significant implications for the prevention and treatment of this condition.

#### 4.3.1 The NLRP3 inflammasome participates in renal IRI via distinct inflammatory signaling pathways

The kidney is endowed with an exceptionally rich blood supply and plays a vital role in maintaining electrolyte homeostasis and excreting metabolic waste. To support these functions, renal tissues, particularly tubular epithelial cells, possess a high mitochondrial density, rendering them highly sensitive to hypoxia and energy depletion. Renal IRI is often unavoidable during kidney transplantation. During IRI, the kidney mounts a robust inflammatory response, with its morphology, hemodynamics, epithelial cells, and endothelial cells all affected to varying degrees (246). Under brief ischemic conditions, endothelial cells and platelets can exert protective effects by inducing coagulation dysfunction and promoting leukocyte activation; however, prolonged or severe ischemia leads to cellular injury and death (247). The NLR family is intimately linked to multiple renal pathophysiological processes. Upon stimulation, activation of innate immune signaling pathways, such as NF-κB and inflammasome-associated pathways, initiated by NLR and related receptor molecules can alter metabolic patterns in renal cells and modify the phenotypes of both immune and parenchymal cells. These changes trigger the secretion of diverse inflammatory mediators, ultimately resulting in irreversible renal tissue damage and functional impairment (247). During renal IRI, the NLRP3 inflammasome contributes to inflammatory responses through two major pathways: the canonical pathway, which depends on Caspase-1 to exert pro-inflammatory effects, and the non-canonical pathway, which primarily relies on Caspase-4, Caspase-5, or Caspase-11 (247).

#### 4.3.2 The NLRP3 inflammasome participates in renal IRI via the canonical inflammatory signaling pathway

The assembly of the NLRP3 inflammasome plays a pivotal role in renal IRI, contributing to multiple pathophysiological processes including tissue damage, inflammatory responses, and fibrosis. In exploring the mechanisms by which necrotic injury is converted into inflammation *in vivo*, numerous studies have demonstrated that the NLRP3 inflammasome primarily exerts its pro-inflammatory effects in renal IRI through the canonical inflammatory signaling pathway (248). Researchers utilizing NLRP3 inflammasome-deficient mice to establish IRI models observed, following reperfusion, enhanced proliferation of tubular epithelial cells, reduced tubular necrosis and apoptosis, and subsequently re-aggregated tubular epithelial cells—indicating that the NLRP3 inflammasome impedes the repair response after IRI (248). Extensive evidence indicates that the NLRP3 inflammasome mediates renal IRI predominantly through the canonical pathway dependent on Caspase-1. Wen et al. (58) found that NLRP3 inflammasome activation was elevated in renal IRI; in wild-type mice subjected to simulated IRI, expression levels of NLRP3 and ASC were upregulated. Moreover, IRI promoted the maturation and secretion of Caspase-1, IL-1β, and IL-18. In contrast to wild-type IRI mice, NLRP3 inflammasome-deficient IRI mice exhibited milder renal pathological damage, with significantly suppressed levels of serum creatinine, blood urea nitrogen, urinary neutrophil gelatinase-associated lipocalin (NGAL), and inflammasome activation. Furthermore, the study revealed that ROS responsible for activating the NLRP3 inflammasome during renal IRI are generated by damaged mitochondria, and that ROS induce NLRP3 inflammasome activation via direct interaction with TXNIP. Iyer et al. demonstrated that NLRP3 inflammasome deficiency protects animals from lethal renal ischemic injury: During ischemic acute tubular necrosis, the NLRP3 inflammasome drives excessive acute inflammation, thereby contributing to IRI-induced renal dysfunction and lethal tubular injury (237). Comparisons between NLRP3-deficient and wild-type mice revealed statistically significant differences in survival rates, blood urea nitrogen, serum creatinine, and neutrophil infiltration. Additionally, Kim et al. reported that, compared with wild-type mice, NLRP3 inflammasome-deficient mice exhibited reduced levels of blood urea nitrogen, serum creatinine, acute tubular necrosis scores, and apoptosis scores, further confirming the protective effect conferred by NLRP3 inflammasome deficiency against ischemic AKI (238).

#### 4.3.3 The NLRP3 inflammasome participates in renal IRI via non-canonical inflammatory signaling pathways

Studies have shown that NLRP3 deficiency ameliorates renal IRI in mice, whereas ASC deficiency confers less pronounced protection against lethal renal ischemic injury (249). This suggests that, within the kidney, components of the NLRP3 inflammasome other than ASC may independently contribute to damage signaling. A recent study demonstrated that the NLRP3 inflammasome can induce pyroptosis independently of Caspase-1 and gasdermin D (GSDMD) (250). Moreover, LPS and oxidized phospholipids can directly bind and activate Caspase-11, Caspase-4, and Caspase-5, triggering inflammasome assembly even in macrophages lacking canonical adaptor proteins (251). Shigeoka et al. (252) reported that NLRP3 deficiency impaired IL-1β and IL-18 production, yet blockade of IL-1β and IL-18 did not significantly attenuate cellular injury, suggesting that in renal tubular epithelial cells, NLRP3 can initiate damage responses independent of canonical inflammasome components, pro-inflammatory cytokines, or chemokines. Kim et al. (63) further demonstrated that under hypoxic conditions, ASC and NLRP3 showed no significant co-localization; instead, NLRP3 relocalized to mitochondria in renal tubular epithelial cells even in the absence of ASC or caspase-1, where it modulated mitochondrial damage and apoptosis during ischemia–hypoxia via the mitochondrial antiviral signaling protein (MAVS), thereby regulating renal ischemia–reperfusion injury (IRI) independently of ASC or caspase-1.

In summary, renal IRI and the pathogenesis of numerous inflammatory diseases are closely linked to the excessive activation of the NLRP3 inflammasome, suggesting that NLRP3 represents a potential therapeutic target for these conditions. However, clinically approved pharmacological agents specifically targeting the NLRP3 inflammasome remain lacking. Recent studies have identified several strategies capable of downregulating NLRP3 expression or inhibiting NLRP3 inflammasome assembly. Compounds such as nodakenin, allopurinol, epoxyeicosatrienoic acids (EETs), and protein C activators have been shown to directly suppress NLRP3 inflammasome activation (236, 253–255). Inhibition of channel or receptor activity, including Pannexin-1 and P2X4, can also attenuate NLRP3 inflammasome activation (53, 256). Furthermore, microRNAs (miRNAs), such as miR-223, miR-9, and miR-155, have been reported to downregulate NLRP3 expression by directly targeting the NLRP3 gene (257, 258). Recently, researchers discovered that Leishmania parasite infection induces fine-tuned transcriptional responses via macrophage histone H3 modifications, which can suppress activation of both NF-κB and the NLRP3 inflammasome (259). These findings collectively suggest that multiple therapeutic avenues exist to inhibit NLRP3 inflammasome activity for the treatment of renal IRI; suppressing its overactivation may represent a viable strategy for preventing and managing renal IRI. Future research should further elucidate the specific signaling events and molecular mechanisms by which the NLRP3 inflammasome drives renal IRI in humans, thereby providing novel insights and therapeutic approaches for clinical intervention.

## 5 Pharmacological targeting of the NLRP3 inflammasome in kidney diseases

Numerous biological inhibitors targeting the NLRP3 inflammasome have been developed to date (**Table 1**); however, their efficacy and safety profiles in the context of kidney diseases remain to be fully established.

**Table 1:** Research progress of drugs targeting the NLRP3 inflammasome in kidney applications.

Drug	Target	Mechanism	Clinical trial status	Kidney disease	Main adverse effects and reference
MCC950	NLRP3	Blocking NLRP3-induced ASC oligomerization	Preclinical research	Diabetic nephropathy, hypertensive nephropathy, contrast-induced nephropathy, kidney injury induced by cisplatin and sepsis	Hepatotoxicity (67, 143, 260–265)
Tranilast	NLRP3	Enhances NLRP3 ubiquitination; binds to NACHT and inhibits NLRP3-NLRP3 interactions	Clinical application	Diabetic nephropathy	NA (266–269)
β-Hydroxybutyrate	NLRP3	Inhibits K^+^ efflux and reduces ASC oligomerization and speck formation	Preclinical research	Hyperoxalate-induced tubular injury	NA (270)
CY-09	NLRP3	Binds to the ATP-binding motif of the NACHT domain and inhibits it	Preclinical research	Ischemia–reperfusion acute kidney injury	NA (142, 271)
VX-740/765	NLRP3	ATPase activity; caspase-1 selectively inhibits caspase-1	Preclinical research	NA	Hepatotoxicity (272)
AZD9056	P2X7	Antagonizes P2X7	Phase II	NA	NA (273)
Brilliant Blue G	P2X7	Selective P2X7 antagonism	Preclinical research	Hypertensive nephropathy, lupus nephritis	NA (274–276)
Glibenclamide	K^+^ channels	ATP-sensitive K^+^ channel inhibitor	Clinical application	Chronic kidney disease	Glucose metabolism abnormalities (277, 278)

### 5.1 Drugs targeting NLRP3

MCC950 is currently the most potent and highly selective NLRP3 inhibitor reported to date (261) (**Table 1**). As a small-molecule diarylsulfonylurea compound, MCC950 selectively inhibits NLRP3 inflammasome activation by blocking ASC oligomerization (279). In diabetic nephropathy models, both *in vivo* and *in vitro*, MCC950 attenuates glomerular basement membrane thickening, podocyte injury, and renal fibrosis by suppressing the NLRP3/caspase-1/IL-1β signaling axis (143, 260). In hypertensive mice, MCC950 reduces blood pressure and proteinuria while alleviating renal inflammation and fibrosis (261). In crystal-induced nephropathy, MCC950 ameliorates renal fibrosis by inhibiting inflammasome activation and the production of IL-1β and IL-18 (262). Moreover, MCC950 mitigates podocyte injury in models of obesity-associated glomerulopathy and lupus susceptibility, as well as kidney damage induced by sepsis (67, 263, 264). Additionally, MCC950 improves cisplatin-induced renal dysfunction by reducing oxidative stress and inflammation, thereby alleviating tubular injury and fibrosis (261). Despite these promising findings and its advantages as a small molecule with high specificity, the safety profile of MCC950 for treating kidney diseases remains to be fully established. Notably, a phase II clinical trial of MCC950 for rheumatoid arthritis was halted due to hepatotoxicity concerns (265). Tranilast, an analog of a tryptophan metabolite and a traditional anti-allergic drug, has recently been shown to directly target and inhibit NLRP3 activity (266). Tranilast enhances NLRP3 ubiquitination by binding to its NACHT domain, thereby disrupting NLRP3–NLRP3 interactions and preventing inflammasome assembly and activation (266, 267). Tranilast reduces oxidative stress (280), inhibits mast cell infiltration (277), suppresses extracellular matrix (ECM) deposition (278), attenuates epithelial–mesenchymal transition (EMT) (281), decreases proteinuria (282), and mitigates tubulointerstitial fibrosis (283), playing a critical role in halting the progression of renal fibrosis. It also ameliorates nephrotoxicity induced by cyclophosphamide and cyclosporine (268, 269). Although not yet approved for kidney diseases, tranilast is already clinically used for various inflammatory conditions and is generally well-tolerated with minimal adverse effects (266). Preclinical studies demonstrate its substantial therapeutic potential in animal models of kidney disease, warranting future clinical investigations into its efficacy and safety across diverse renal pathologies. β-Hydroxybutyrate (BHB), identified in recent years as an endogenous inhibitor of the NLRP3 inflammasome, prevents K^+^ efflux and suppresses ASC oligomerization and speck formation (270). Studies show that BHB alleviates tubular injury in mice fed a high-oxalate diet and promotes a phenotypic switch in macrophages from a pro-inflammatory to an anti-inflammatory state. These findings suggest that BHB may mitigate renal inflammation and holds promise as a potential therapeutic agent for kidney-related diseases via NLRP3 inflammasome inhibition. CY-09 is a direct NLRP3 inflammasome inhibitor identified to date. It binds to the ATP-binding motif within the NACHT domain of NLRP3 and inhibits its ATPase activity, thereby blocking inflammasome assembly and activation (142). CY-09 has demonstrated efficacy in animal models of various diseases, including obesity and associated non-alcoholic fatty liver disease (284), type 2 diabetes (142), and epilepsy (285). However, reports on its application in kidney diseases remain limited. To date, CY-09 has only been shown to ameliorate renal dysfunction induced by ischemia-reperfusion injury (271).

### 5.2 Drugs targeting caspase-1

VX-740 and VX-765 are peptidomimetic prodrugs that inhibit caspase-1. Both compounds advanced to Phase II clinical trials for the treatment of psoriasis, arthritis, and epilepsy; however, their development was discontinued due to hepatotoxicity concerns (272). To date, no clinical trials evaluating the efficacy or safety of these agents in kidney diseases have been reported. 

### 5.3 Drugs targeting P2X7

AZD9056 is the first P2X7 receptor antagonist to have successfully entered clinical trials, demonstrating significant efficacy in phase IIa trials for rheumatoid arthritis. In addition to AZD9056, other P2X7 inhibitors, such as CE-224,535, have also advanced into clinical development (286). However, no studies to date have evaluated their application in the treatment of kidney diseases. Brilliant Blue G (BBG) is a selective P2X7 receptor antagonist. Experimental evidence indicates that BBG can attenuate inflammation and fibrosis (274, 275). In animal models, BBG has been shown to ameliorate renal injury in Dahl salt-sensitive rats and LN mice (276). Mechanistically, BBG suppresses macrophage and fibroblast infiltration, reduces the expression of inflammatory cytokines and collagen, inhibits apoptosis, and promotes regeneration of renal tubular epithelial cells (287). Nevertheless, the safety profile and clinical efficacy of BBG in human kidney diseases remain to be investigated in controlled clinical trials.

### 5.4 Drugs targeting K^+^ channels

**Figure 5:**
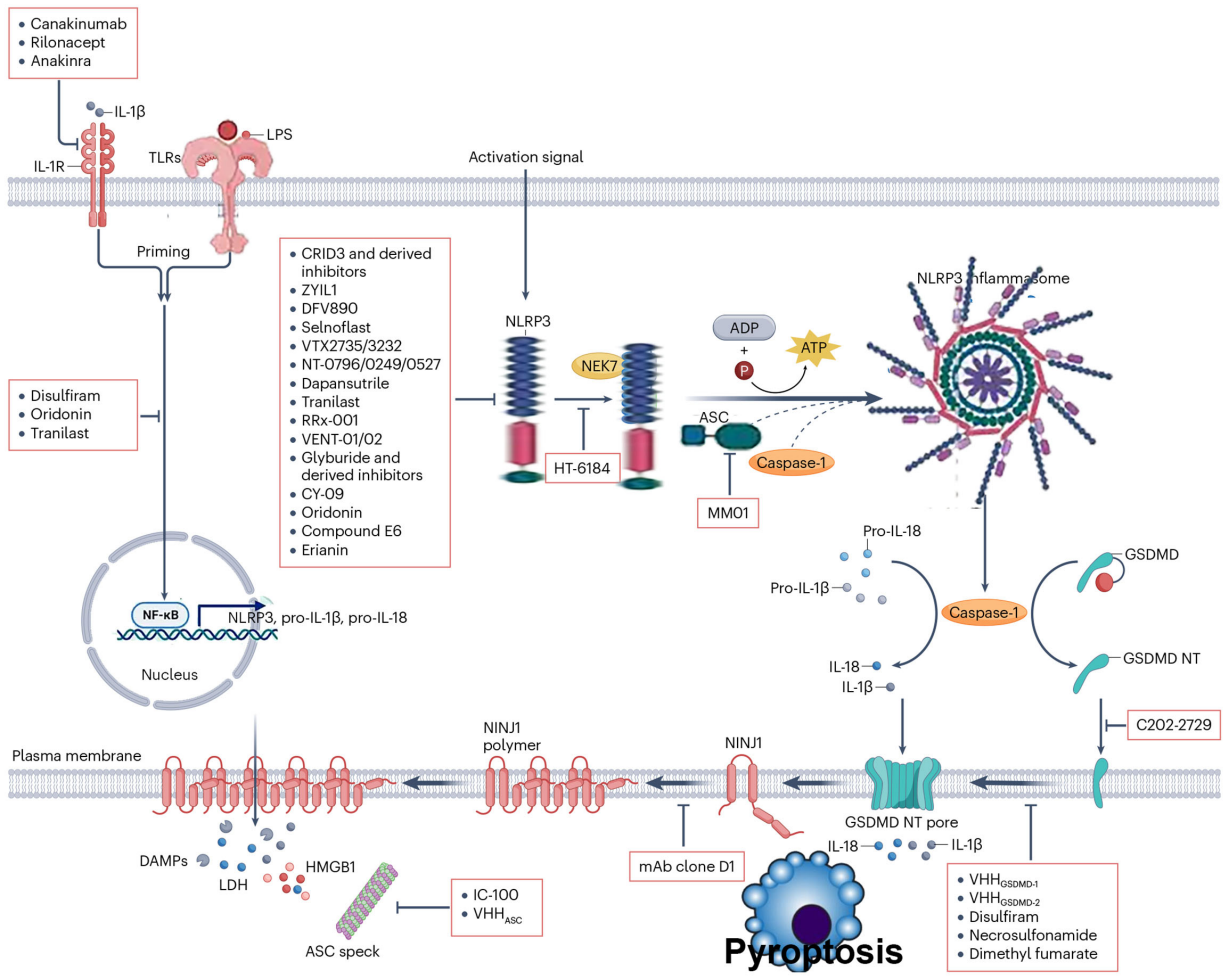
Therapeutic targets and inhibitors of the NLRP3 inflammasome include clinically approved biologics such as canakinumab, rilonacept, and anakinra, which inhibit inflammation by blocking interleukin-1β from binding to the interleukin-1 receptor on effector cells, acting downstream of inflammasome activation. Compounds such as disulfiram, oridonin, and tranilast inhibit inflammasome signaling at various stages, including the initial activation step. The discovery that sulfonylurea-containing inhibitors CRID3 and glyburide selectively inhibit the NLRP3 activation step has paved the way for second-generation clinical-grade NLRP3 inhibitors with enhanced potency and improved pharmacological profiles, such as ZYIL1, DFV890, and selnoflast. VTX3232, VTX2735, NT-0796, and NT-0249 are additional NLRP3 inhibitors in clinical development whose structures remain undisclosed. HT-6184 modulates NEK7 in addition to inhibiting NLRP3. The anti-allergy drug tranilast and the anticancer candidate RRx-001 may not be specific to NLRP3, as they exhibit additional immunomodulatory activities. Other NLRP3 inhibitors in preclinical development include VENT-01, VENT-02, and NT-0527. CRID3 reversibly binds to the NACHT domain of NLRP3. Other compounds proposed to inhibit NLRP3 via binding to its NACHT domain include CY-09, tranilast, and the covalent NLRP3 inhibitors shikonin and oridonin. Compound E6, a novel oridonin derivative, exhibits greater potency and selectivity. Additional inflammasome targets acting downstream of NLRP3 and other inflammasome sensors include apoptosis-associated speck-like protein containing a CARD (ASC), GSDMD, and nerve injury-induced protein 1 (NINJ1). The small-molecule ASC inhibitor MM01 suppresses ASC oligomerization, whereas the biologics IC-100 and the VHHASC nanobody target extracellular ASC specks. C202–2729 is thought to non-covalently bind and inhibit translocation of the GSDMD N-terminal (NT) domain to the plasma membrane. The FDA-approved drugs disulfiram and dimethyl fumarate, as well as the necroptosis inhibitor necrosulfonamide, inhibit GSDMD through covalent modification; however, these molecules exhibit polypharmacological properties. The GSDMD-targeting nanobodies VHHGSDMD-1 and VHHGSDMD-2 inhibit GSDMD polymerization in the plasma membrane without interfering with membrane insertion of the GSDMD NT domain. The antagonistic NINJ1 monoclonal antibody (mAb) clone D1 inhibits NINJ1 polymerization and pyroptotic lysis downstream of GSDMD NT membrane pores. DAMP, damage-associated molecular pattern; HMGB1, high-mobility group box 1; LDH, lactate dehydrogenase; LPS, lipopolysaccharide; NF-κB, nuclear factor kappa B; TLR, Toll-like receptor.

Lamkanfi et al. (232) demonstrated that glibenclamide (glyburide), by binding to ATP-sensitive K^+^ channels, can inhibit NLRP3 inflammasome activation. As an NLRP3 inflammasome inhibitor, glibenclamide has been shown to attenuate adenine-induced CKD and renal fibrosis in rats (288). However, as a sulfonylurea antidiabetic agent, its clinical utility beyond glycemic control is limited by the risk of hypoglycemia and glucose metabolism disturbances (289). Therapeutic targets and inhibitors of the NLRP3 inflammasome are detailed in **Figure 5** below.

In summary, significant progress has been made in elucidating the functional roles of the NLRP3 inflammasome in kidney diseases. The NLRP3 inflammasome is now recognized as a key contributor to the initiation and progression of multiple renal disorders; however, its detailed mechanistic actions, clinical translatable, and particularly its inflammasome - independent functions remain incompletely understood and are still in their infancy. A deeper understanding of its associated signaling pathways, regulatory networks, and pathophysiological significance will be instrumental in formulating novel strategies for the prevention and treatment of kidney diseases. Compared with currently employed large - molecule biologics (290), small - molecule inhibitors that directly target the NLRP3 inflammasome offer distinct advantages, including higher target specificity, lower manufacturing costs, and reduced toxicity due to lower effective dosing requirements — thus demonstrating considerable therapeutic promise. Several investigational agents targeting the NLRP3 inflammasome or its downstream effectors have already shown encouraging results in non - renal diseases (291); however, research evaluating their efficacy specifically in kidney pathologies remains limited. These agents may hold substantial potential for treating both AKI and CKD[ (292). Nevertheless, translating promising preclinical findings into clinical applications will require substantial time and effort, and the efficacy and safety profiles of these compounds in renal disease contexts remain to be definitively established.

## 6 Discussion, perspectives, and challenges: the translational road to targeting the NLRP3 inflammasome in kidney disease

### 6.1 From mechanistic consensus to clinical heterogeneity: the “double-edged sword” nature of NLRP3 in kidney disease

**Figure 6:**
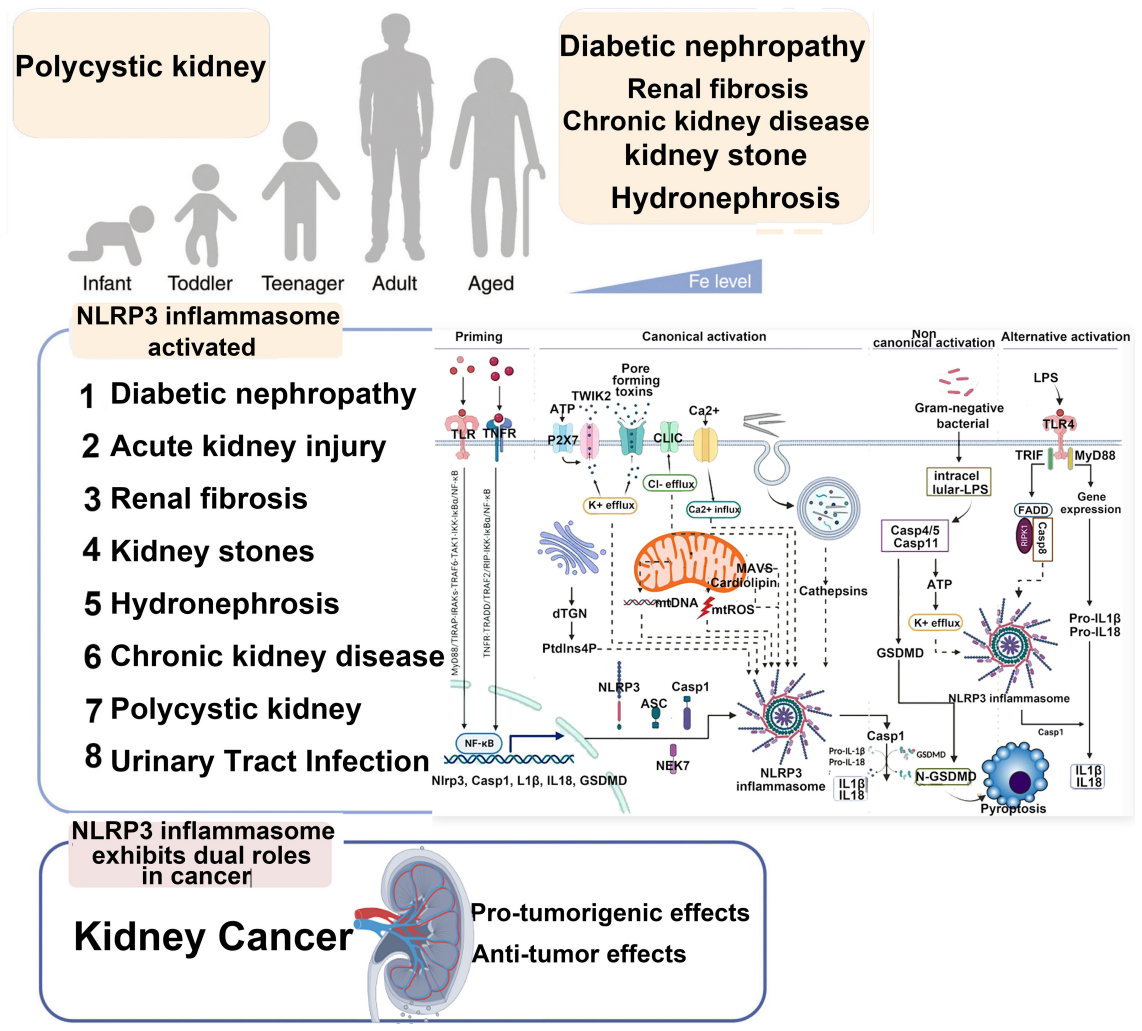
The NLRP3 inflammasome is involved in kidney diseases throughout the human lifespan.

Although a broad scientific consensus supports the pathogenic role of the NLRP3 inflammasome in kidney diseases, its activation exhibits remarkable functional heterogeneity across disease stages, cell types (e.g., tubular epithelial cells, podocytes, macrophages, dendritic cells), and even patient subpopulations. For instance, during the early phase of AKI, moderate NLRP3 activation may exert protective immune surveillance functions by facilitating the clearance of necrotic cellular debris; conversely, in CKD, persistent NLRP3 activation drives irreversible fibrosis and functional decline. This “time–space–dose”–dependent duality underscores that blunt “inhibition of NLRP3” is not a universal therapeutic solution—instead, context-specific modulation strategies must be developed to match disease stage, cellular microenvironment, and individual patient profiles. Adding further complexity, NLRP3 does not operate in isolation but is embedded within a broader “inflammatory network,” engaging in extensive crosstalk with pathways such as TLR4, NF-κB, cGAS-STING, RIPK3-MLKL, and the autophagy–lysosomal system. For example, in DKD, high glucose–induced mitochondrial ROS not only activates NLRP3 but also concurrently suppresses PINK1/Parkin-mediated mitophagy, thereby establishing a self-amplifying feedback loop linking “inflammation–metabolic stress–organelle damage”. Consequently, future research must transcend the ‘single-target’ paradigm and embrace systems pharmacology approaches to identify critical nodal regulators (e.g., NEK7, TXNIP, Gasdermin D) or engineer multitarget combinatorial interventions that simultaneously disrupt pathogenic circuits while preserving homeostatic functions (**Figure 6**).

### 6.2 Clinical translation of small-molecule inhibitors: bridging the gulf from “laboratory star” to “clinical reality”

Currently, NLRP3-specific inhibitors, exemplified by MCC950, have demonstrated striking renoprotective effects in rodent models, including attenuation of proteinuria, suppression of interstitial fibrosis, and improvement of renal function. Nevertheless, their journey toward clinical application remains fraught with formidable obstacles:

1. Species disparities and model limitations: The majority of preclinical studies rely on acute or subacute injury models (e.g., cisplatin-induced AKI, UUO]), which inadequately recapitulate the protracted, decades-long progression characteristic of human CKD. Furthermore, significant interspecies differences exist between rodents and humans in NLRP3 expression patterns, IL-1β cleavage efficiency, and susceptibility to pyroptosis—leading to inaccurate extrapolation of drug efficacy and potential clinical failure.
2. Tissue targeting and bioavailability bottlenecks: The kidney’s structural heterogeneity, which spans the cortex, medulla, glomeruli, and tubules, poses a major pharmacological challenge. Most existing small molecules are administered systemically and often fail to accumulate at therapeutic concentrations within pathologically relevant microenvironments, such as the hypoxic medulla or fibrotic interstitium. Overcoming these pharmacokinetic barriers demands innovative delivery platforms, including tubule- or podocyte-targeted nanocarriers and ligand–drug conjugates engineered to bind brush border receptors in the proximal tubule—thereby enhancing renal specificity and minimizing off-target exposure.
3. Safety concerns and off-target effects: NLRP3 fulfills essential physiological roles in host defense, including antifungal and anti-intracellular bacterial immunity. Long-term systemic inhibition may therefore compromise immune surveillance and increase infection risk. Although OLT1177 (dapansutrile) has demonstrated favorable safety profiles in clinical trials for gout and osteoarthritis, longitudinal immunological monitoring data in renal patient cohorts, particularly those with advanced CKD or immunosuppressive comorbidities, are still lacking. Future therapeutic design must prioritize conditional activation strategies, such as pH-sensitive or ROS-responsive prodrugs that release active compounds only in inflamed microenvironments, or localized delivery systems (e.g., perirenal injectables or implantable microdevices) that restrict pharmacological action to the kidney while sparing systemic immunity.

### 6.3 NLRP3-targeted therapy in the era of precision medicine: from “one-size-fits-all” to “patient stratification”

Current clinical trial designs largely overlook the profound heterogeneity in patients’ inflammatory phenotypes. There is an urgent need to establish a comprehensive biomarker signature of NLRP3 activation to guide patient selection, predict therapeutic response, and monitor dynamic changes during treatment. Promising candidate biomarkers include the following: (1) plasma or urine biomarkers: IL-18, caspase-1 p20 fragment, and N-terminal fragment of gasdermin D; (2) peripheral blood mononuclear cell (PBMC) assays: NLRP3 mRNA expression levels or frequency of ASC speck formation; (3) renal biopsy-based profiling: immunohistochemical scoring of NLRP3, ASC, and caspase-1 protein expression, or spatial transcriptomic mapping of inflammatory “hotspot” regions. By integrating multi-omics data (e.g., intrarenal immune microenvironment revealed by single-cell RNA-seq, plasma proteome, metabolome), an “NLRP3 Inflammatory Index” can be constructed to enable pre-therapeutic risk stratification. For example, patients with a high “Pyroptosis Index” may be more sensitive to gasdermin D inhibitors, whereas those with an “IL-1β-dominant” profile may be better suited for IL-1 receptor antagonists (e.g., anakinra) or upstream NLRP3 inhibitors.

### 6.4 Future frontiers: beyond canonical NLRP3—exploring next-generation intervention strategies

1. Targeting NLRP3 assembly with “molecular glues” and allosteric modulators

Current inhibitors predominantly act on the NLRP3 NACHT domain (e.g., MCC950), but newly identified allosteric pockets (e.g., the HD2 subdomain of NLRP3) and protein–protein interfaces (e.g., NLRP3–NEK7, ASC–ASC) offer opportunities to develop highly selective “molecular glues” or PROTAC degraders.

2. Epigenetic and metabolic reprogramming to regulate NLRP3

Histone modifications (e.g., H3K27ac), non-coding RNAs (e.g., miR-223, lncRNA NEAT1), and metabolites (e.g., succinate, itaconate) can modulate NLRP3 transcription and activation. Indirect regulatory strategies targeting epigenetic enzymes (e.g., BET inhibitors, HDACi) or metabolic enzymes (e.g., IRG1/itaconate pathway) may enable more durable and safer inflammatory silencing.

3. Prospects for gene editing and cell-based therapies

In hereditary kidney diseases (e.g., familial Mediterranean fever-associated renal amyloidosis), CRISPR-Cas9–mediated NLRP3 gene editing or transplantation of iPSC-derived, genetically corrected renal cells may offer “one-time curative” solutions. Although still conceptual, these approaches warrant forward-looking investment.

### 6.5 Challenges and unresolved mysteries: key scientific questions urgently requiring breakthroughs

- Does NLRP3 exert pro-regenerative functions in renal progenitor or repair-associated cells? Could its inhibition impair tissue regeneration?
- How is the dynamic crosstalk—or “death dialogue”—among pyroptosis, apoptosis, and necroptosis balanced during kidney injury? Does a therapeutic window exist for modulating “cell death modality switching”?
- Within the gut–kidney axis, how do microbial metabolites (e.g., TMAO, butyrate) remotely regulate intrarenal NLRP3 activation? Can probiotics or dietary interventions be harnessed to therapeutically modulate the “gut–renal immune axis”?
- How do sex differences and hormonal regulation (e.g., estrogen-mediated suppression of NLRP3) influence therapeutic responses? Should clinical trials be stratified by sex?

### 6.6 Concluding remarks: toward a five-dimensional translational framework—”mechanism–target–patient–delivery–monitoring”

Targeting NLRP3 for kidney disease therapy has transitioned from the stage of “proof-of-concept” to the critical phase of “precision translation”. Future success will depend not only on the development of superior small-molecule chemical entities but also on the establishment of an integrated five-dimensional translational medicine framework encompassing deep mechanistic dissection, precise patient stratification, intelligent drug delivery systems, dynamic efficacy monitoring, and real-world validation. Only through such a holistic approach can the brilliant “light of the inflammasome” observed in the laboratory be truly transformed into a “clinical torch” illuminating the path to improved outcomes for millions of patients suffering from kidney disease.
